# Direct Laser Writing of Polymer Nanocomposites for Tunable Structural Color

**DOI:** 10.1002/adma.202504116

**Published:** 2025-07-09

**Authors:** Teodora Faraone, Jing Qian, Srikanth Kolagatla, A. Louise Bradley, Larisa Florea, Colm Delaney

**Affiliations:** ^1^ School of Chemistry and AMBER The SFI Research Center for Advanced Materials and BioEngineering Research Trinity College Dublin Dublin 2 Dublin D02PN40 Ireland; ^2^ School of Physics and AMBER The SFI Research Center for Advanced Materials and BioEngineering Research Trinity College Dublin Dublin 2 Dublin D02PN40 Ireland

**Keywords:** direct laser writing, nanocomposites, photonic‐crystal, self‐assembly

## Abstract

Self‐assembled colloidal particles offer a means of manipulating light, giving rise to some of the most brilliant structural colors. Conventionally, changing the reflection band of colloidal crystal assemblies has required the synthesis of different‐sized nanoparticles or multiple fabrication steps. This is not a means conducive to fine‐tuning structural color. Herein, it is shown that, by combining nanocomposite photoresists, self‐assembly, and direct laser writing, it is possible to achieve high‐resolution fabrication and precise placement of nanoparticles, combined with highly tunable structural color using one composition and one particle size. Confinement of polymerization brought by direct laser writing allows for fine control over a series of fabrication parameters, such as slicing and hatching distances, thereby enabling control of the inter‐particle distance in a nanocomposite. This is key to generating microstructures that exhibit a wide gamut of color that traverses the visible spectrum. Finite‐difference time‐domain simulations are further used as a means of understanding the structural modifications that control color variation. The method described herein is suitable for fine‐tuning of structural color in self‐assembled systems, and is applicable to a wide range of materials.

## Graphical abstract

Herein, direct laser writing is used to polymerize self‐assembled nanocomposite materials. Using fabrication parameters, the interparticle spacing is finely tuned beyond the thermodynamically stable state. This is exploited to generate wide gamut structural color in water. Numerical simulations are used to support this, to enable the use of scattering spectra to predict volume fraction and interparticle spacing on the nanoscale.

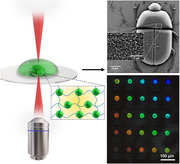

## 1 Introduction

Since the discovery of structural color in nature by Robert Hooke in 1665,^[^ ^1^ ^]^ the development of synthetic materials displaying structural color has been documented in physics, chemistry, and materials science. In recent years, these materials have found applications in photonics,^[^ ^2^ ^]^ biomedicine,^[^ ^3^, ^4^ ^]^ encryption,^[^ ^5^ ^]^ and environmental science.^[^ ^6^ ^]^ Various synthetic approaches have been developed to emulate naturally occurring structural colors, such as multilayer films,^[^ ^7^ ^]^ diffraction gratings,^[^ ^8^, ^9^, ^10^ ^]^ and colloidal crystal assemblies,^[^ ^11^, ^12^ ^]^ which are usually defined by the dimensionality of the periodic array. Submicron‐feature periodicity, combined with a change in refractive index, has been exploited to relay dynamic structural color at the macroscale in a multitude of polymeric materials, including stimuli‐responsive hydrogels. This is illustrated in the work by Wen et al.,^[^ ^10^ ^]^ in which flexible, solvent and temperature‐responsive poly(*N‐isopropyl acrylamide*) (PNIPAM) gratings of a periodic 1D groove structure were achieved using a template, resulting in brilliant angle‐dependent and temperature‐responsive colors. The phenomenon of structural color has also been translated into 2D materials and structures, as demonstrated by Zhang et al.,^[^ ^13^ ^]^ where structural color was achieved using a nanoindenter combined with ultrafast elliptical vibration to create pyramid‐shaped nanostructures on metallic surfaces. 3D arrangements displaying structural coloration often employ the use of synthetic colloidal crystals. This has been accomplished using a variety of materials, including inorganic particles such as silica nanoparticles,^[^ ^14^, ^15^, ^16^ ^]^ block copolymers,^[^ ^17^ ^]^ natural derivatives such as dopamine,^[^ ^18^ ^]^ melanin,^[^ ^19^, ^20^ ^]^ or cellulose,^[^ ^21^, ^22^, ^23^ ^]^ and polymer nanoparticles of various compositions,^[^ ^11^, ^12^, ^24^ ^]^ including styrene latex‐based particles.^[^ ^25^, ^26^ ^]^ This phenomenon has been further exploited by combining it with stimuli‐responsive materials to endow a dynamic response, such as the work reported by Liao et al.^[^ ^27^ ^]^ In their work, digital light processing (DLP) was used to create 3D structures displaying temperature‐dependent colors, using a composite colloidal photonic crystal ink containing highly charged elastic polymer nanoparticles and temperature‐responsive polymers. In the work by Lee et al.,^[^ ^15^ ^]^ non‐close‐packed arrays of silica particles were used in an elastomeric polymer matrix to yield reversible, strain‐dependent structural colors. Another recent example of structural color for stimuli‐response was demonstrated by Smirnov et al.,^[^ ^28^ ^]^ to create magnetically responsive color changes. These colors were tuned by manipulating an external magnetic field that controlled the particle assembly, and thus the reflected color.

Emulation of structural color using colloidal particles is highly dependent on inter‐particle distance, and thus experimental means of producing nanoparticle assemblies with controlled and tunable spacing are particularly important. Controlling inter‐particle distance in a near close‐packed 3D superlattice is challenging, and most common strategies involve synthetic approaches to produce monodispersed nanoparticles (NPs) of variable size or NP functionalisation to control assembly. Once assembled in the 3D superlattice, in most cases face‐centered cubic (fcc) or body‐centered cubic (bcc), the lattice constant is defined and remains unchanged unless the host matrix itself is responsive.^[^ ^15^, ^27^, ^28^ ^]^ Finding means of precisely altering inter‐particle distance in the 3D superlattice post‐self‐assembly offers access to spacings and periodicities currently precluded by the thermodynamics of self‐assembly, which can prove significantly beneficial for the advancement of self‐assembled NP systems in photonics, plasmonics, biosensing, and data storage.

Herein, we demonstrate the use of polymer nanocomposites for the realization of high‐resolution microstructures comprising ordered NP arrays. This was achieved using direct laser writing (DLW) by two‐photon polymerization (2PP) in polymer nanocomposite photoresists, where variation of fabrication parameters, such as slicing and hatching distance, allow for fine‐tuning of inter‐particle distance. These microstructures display brilliant and tunable wide‐gamut structural color, from near‐UV to near‐IR regions. Using finite difference time domain (FDTD) simulations, we use the stop‐band of the structures to elucidate nanoscale ordering on the nanoscale. Not only does this work offer a route to a library of particles with controllable size and low polydispersity, but it offers a means of synthesising particles which can be easily adapted, to improve colloidal stability and self‐assembly, or to introduce additional stimuli‐response. Therefore, this work presents an adaptable approach for producing hierarchical structures from well‐ordered NP assemblies with precise control over interparticle distance, to tune optical, plasmonic, electronic, and magnetic properties.

## 2 Results and Discussion

Two different sizes of monodisperse polymer NPs (PNP1 – d_hyd_ = 186 ± 3 nm, and PNP2 – d_hyd_ = 160 ± 2 nm) of the same chemical composition, were synthesized via surfactant‐free emulsion polymerization in aqueous media (Figure S1, Supporting Information). Both PNP1 and PNP2 were composed of the hydrophobic methyl methacrylate (MMA) monomer as the main component, and hydroxyethyl acrylate (HEA) as a hydrophilic co‐monomer. 1,6‐hexanediol diacrylate (HDDA) was used as the crosslinker. The change in PNP diameter was achieved by carefully adjusting the molar ratios of HEA and MMA. Smaller HEA mol% wrt. MMA resulted in smaller diameter PNPs (PNP1) and vice versa, where larger HEA mol% resulted in larger diameter PNPs (PNP2) (Table S1, Supporting Information). SEM analysis was used to measure PNP diameter in the dry state, PNP1– 117 ± 12 nm; PNP2 – 100 ± 7 nm, and to confirm spontaneous self‐assembly of the NPs (Figure S2, Supporting Information).

**Figure 1:**
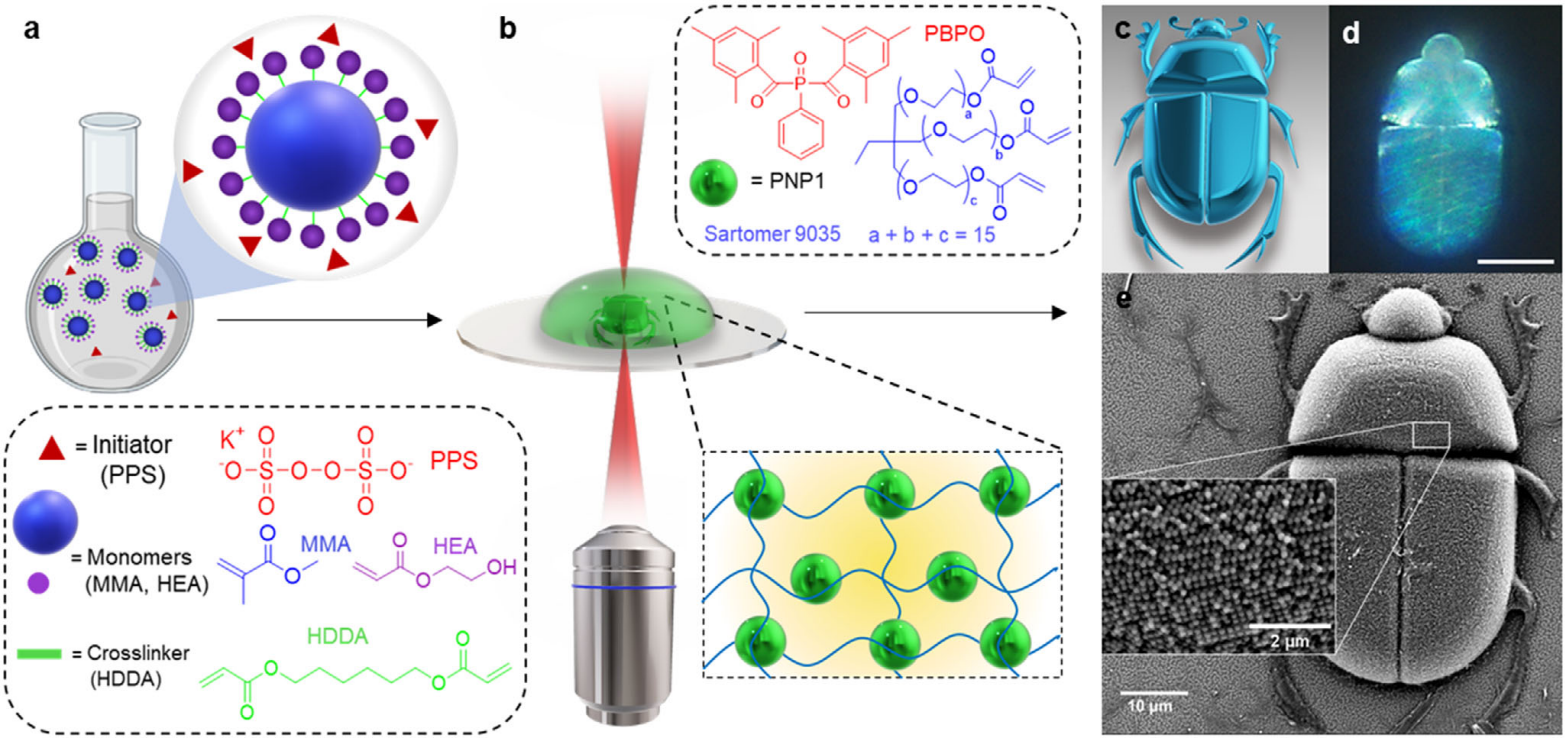
Conceptual illustration of this study. a) Nanoparticle synthesis via emulsion polymerization. b) Schematic representation of the DLW process showing chemical compositions of Photoresist 1 (top) and self‐assembled nanoparticles within (bottom). c) 3D design of a scarab beetle structure. d) Dark‐field optical microscopy image of the structurally colored scarab beetle microstructure fabricated with Photoresist 1 hydrated in deionised (DI) water (scale bar represents 20 µm). e) corresponding SEM picture with magnification insert showing self‐assembled nanoparticles (PNP1).

For the fabrication of microstructures containing PNP1 via DLW, Photoresist 1 was prepared. This was achieved by dispersing PNP1 particles in ethoxylated (15) trimethylolpropane triacrylate (SR 9035). The refractive index of the dry polymerized SR 9035 was measured using an Abbe Refractometer (Shanghai CSOIF CO., LTD) and was found to be 1.4931. The composition was optimised as described in Table S2 (Supporting Information). The optimised photoresist facilitated the self‐assembly of the PNPs. The amount of PNP1 added to the photoresist (45 wt.%) exceeded the threshold volume fraction, Φ_𝑡ℎ_ (defined below in Equation 1). Above this threshold, the solvation layers of the particles are expected to overlap and self‐assemble due to inter‐particle repulsion, leading to the fulfillment of a Bragg condition, therefore resulting in structural color.^[^ ^15^ ^]^

(1) $$\Phi_{\mathrm{th}}=\frac{\pi d_{\mathrm{PNP}}^{3}}{3\sqrt{2}{\left(d_{\mathrm{PNP}}+2t\right)}^{3}}\;$$

where Φ_𝑡ℎ_ represents the threshold volume fraction,d is the average particle diameter (d_PNP_) and *t* is the thickness of the solvation layer. In the case where the average diameter was set to d_PNP1_ = 117 nm, as obtained from the SEM images of PNP1 in the dry state, and 2*t* was set at 69 nm, the threshold volume fraction of PNP1 in Photoresist 1 equals 18.4 vol%. However, in the case of the composite, the surface‐to‐surface separation between two nearest PNPs is likely to be smaller than the diameter of the solvated particle, thereby a larger threshold is practically needed to achieve particle self‐assembly. Furthermore, this threshold volume fraction value was also confirmed by other previous studies for similarly sized particles.^[^ ^29^ ^]^ Therefore, a PNP1 weight percentage value of 45% was chosen for Photoresist 1 (Figure S3, Supporting Information). Phenylbis(2,4,6‐trimethylbenzoyl) phosphine oxide (PBPO) was selected as the photoinitiator, as it shows good solubility in the acrylate monomers, efficient radical formation, and suitability for two‐photon polymerization (2PP) due to its two‐photon absorption (2PA) cross section.^[^ ^30^ ^]^ Photoresist 2 was prepared following the same protocol, by dispersing PNP2 in a monomer cocktail of the same composition (**Figure** **1**; Table S3, Supporting Information). Prior to DLW, a small droplet of the PNP photoresist was placed at the opening of a glass cell and allowed to fill the cell through capillary action (Figure S3, Supporting Information). The cell was then cooled for at least 12 h (7–10 °C), resulting in a vibrantly colored photoresist film (Figure S4, Supporting Information).

Following photoresist preparation, DLW was employed for the fabrication of a range of microstructures based on 3D designs, as described in Figure 1. These were fabricated to demonstrate both the mechanical robustness and the fidelity to the 3D design achieved by using Photoresist 1 (Figure 1; Figure S21, Supporting Information). During fabrication optimisation, the laser dosage (laser power – LP, and scan speed – SS) was varied to ensure successful fabrication of high‐fidelity microstructures. To investigate the optical properties of the material, a 5 × 5 micro‐pillar array (dimensions: d = 30 µm, height = 30 µm) was fabricated using the optimised writing parameters (writing speed – 10000 µm s^−1^ and laser power (LP) 60%) and varying the slicing distance (SL = 0.1 to 0.5 µm) with increments of 0.1 µm along the *z*‐axis and the hatching distance (HD = 0.1 to 0.5 µm) with increments of 0.1 µm along the xy‐axes. These laser dosage parameters were optimized for the photoresist composition and design parameters. Monomer reactivity and concentration, as well as photoinitiator type and concentration, greatly influence the laser dosage needed for optimal structure fabrication.

**Figure 2:**
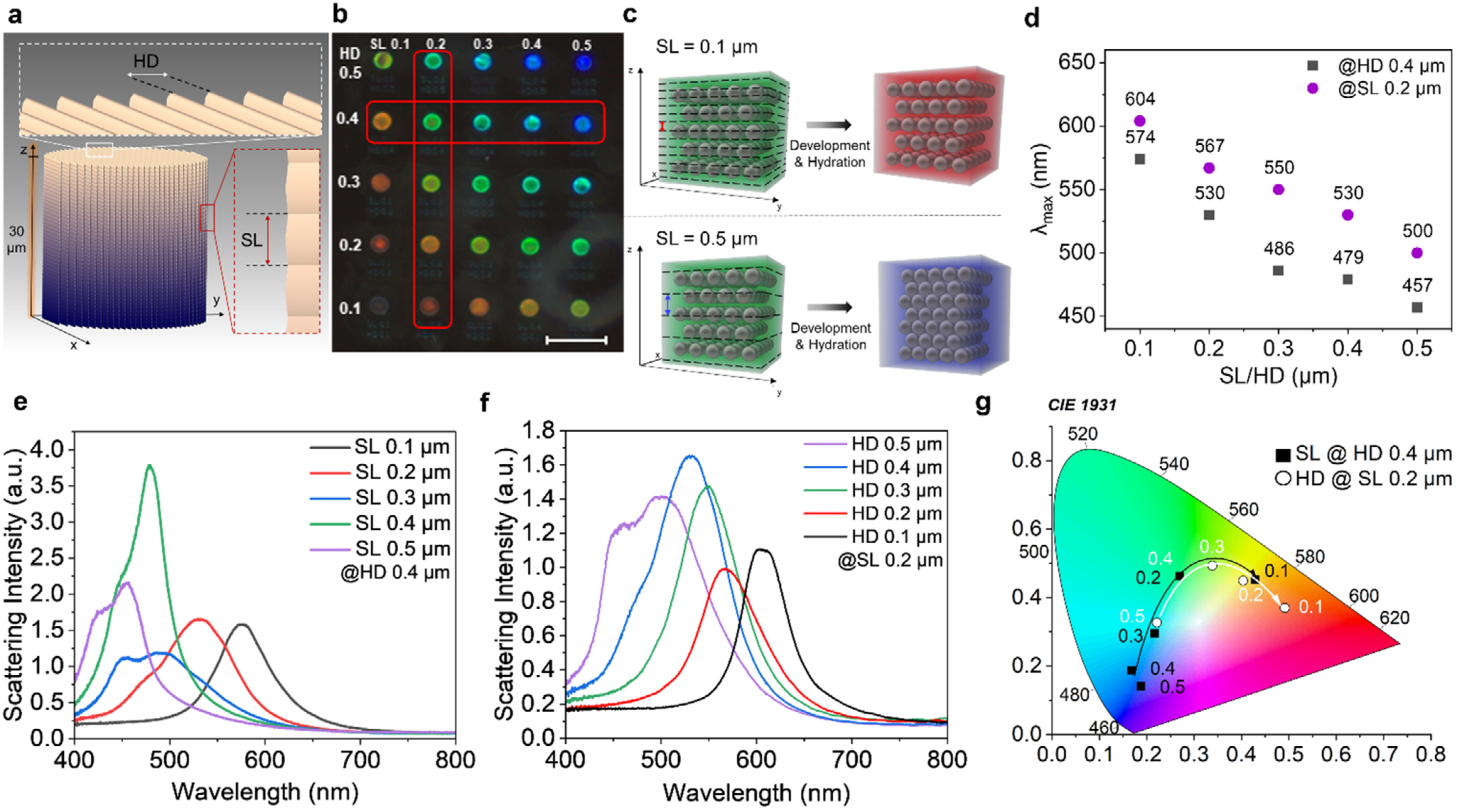
Design of a structurally colored microarray. a) Pillar CAD model (height = diameter = 30 µm) illustrating slicing (SL) and hatching distances (HD). b) Dark field microscopy image of 5 × 5 pillar array hydrated in DI water fabricated with Photoresist 1 (60%, 10000 µm s^−1^). c) Diagram showing a schematic of the PNP collapse in the fabricated composite microstructures upon development and hydration when (top) a small slicing value was employed (i.e SL = 0.1 µm) compared to when (bottom) a larger slicing value was employed (i.e SL = 0.5 µm) during DLW. d) λ_max_ (nm) vs. slicing or hatching distance (µm). e,f) Scattering spectra corresponding to highlighted pillars in panel b. g) Corresponding CIE 1931 diagram. The scale bar in b) represents 100 µm.

The slicing and hatching distances, as illustrated in **Figure** **2**a, define the center‐to‐center voxel distances in the z and x‐y direction, respectively. The rationale behind this array design was to verify whether, by altering the slicing and the hatching distances, the inter‐particle distance would also be affected, thereby altering the reflected color. The change in the reflected wavelength on a well‐ordered structure, from normally incident light, can be predicted by the Bragg‐Snell law combined with the Maxwell‐Garnett approximation (Equation 2).

(2) $$\lambda_{\max}=2d_{\mathrm{int}}\sqrt{\eta_{eff}^{2}-\sin^{2}\alpha }$$

where d_int_ = inter‐layer distance; η_eff_ = effective refractive index of PNP‐polymer nanocomposite; α = angle of incident light (α = 0 in normal reflection).

Post fabrication, the microstructures showed no visible color in the dry state; however, upon hydration, the nanocomposite microstructures exhibited wide gamut reflected color. To correlate reflected wavelength with inter‐particle distance using (Equation 2), a 20% overall expansion of the polymer host matrix (SR 9035) was approximated based on experimentally determined data (Table S21, Supporting Information), allowing for η_eff_ of ≈1.46, which was estimated for the hydrated polymer matrix through the Maxwell‐Garnett approximation. The refractive indices of dry and hydrated polymerized SR9035 films were also measured, with the results presented in Table S6 (Supporting Information). The estimated η_eff_ of ≈1.46 matches the experimental refractive index of the hydrated polymer films. Using the recorded λ_max_ values (Table S7, Supporting Information) for each of the pillars, and the refractive index of the particles’ main component (pMMA = 1.49)^[^ ^31^ ^]^ in (Equation 2), it is possible to obtain predicted values for the corresponding interparticle distances and to relate these to the λ_max_ trend observed (Table S7, Supporting Information). A decrease in the reflected wavelength, which corresponds to decreasing interparticle distance, was obtained with increasing SL and HD distances. This correlates smaller interparticle distances with bigger SL and HD values, rationalised by the collapse of the structure post‐development. Larger SL distances cause a greater collapse of the structure post‐development, placing the particles into closer proximity, i.e., decreasing their interparticle distance (Figure 2c). A similar phenomenon was already observed in the work of Ritacco et al.,^[^ ^32^ ^]^ in which cholesteric liquid crystals (LC) were employed in DLW to obtain structural color. Their results revealed that the fabricated LC composite microstructures shrank after development, thus changing their reflected color from NIR (after fabrication, pre‐development) to red (<1 min post‐development), then orange (30 min. post‐development), and finally cyan (24h post‐development). The authors attributed this color change to a partial collapse of the LC network within the structure (and therefore its subsequent shrinking) caused by a lower degree of polymerization due to lower laser dosage (lower LP and faster scan speed). More recently, the work of Augustine et al.^[^ ^33^ ^]^ on the direct laser writing of silica NP composites (not displaying long‐range order), also reported a similar phenomenon when modulating the slicing parameters only. The same hypothesis can be assumed for the work presented herein, based on the obtained optical and microscopy data. As shown from the optical scattering spectra presented in Figure 2, for both the hatching distance increase at constant slicing value (i.e. column 2, HD = 0.1 to 0.5 µm at SL = 0.2 µm), and vice versa, slicing distance increase at constant hatching distance (i.e. row 2, SL = 0.1 to 0.5 µm at HD = 0.4 µm), a decrease in the reflected wavelength (λ_max_) is generally seen. Inversely, smaller SL and HD parameters, e.g., SL/HD = 0.1 µm, show longer reflected wavelengths, which, as determined by (Equation 2), is due to larger inter‐particle distances. Large SL and HD values produced microstructures prone to enhanced structural collapse, resulting in less homogeneous colors with broader spectral peaks. The corresponding colors, plotted on a CIE diagram, show the wide color gamut that can be observed.

An equivalent of Photoresist 1 was also prepared by replacing PNP1 (117 ± 12 nm) with PNP2 (100 ± 7 nm). This resulted in Photoresist 2, composed of the same ratios of SR9035, PBPO, and PNP2 – 45 wt.% (Table S3, Supporting Information). The same 5 × 5 micropillar design used with Photoresist 1 (Figure 2) was also used for Photoresist 2, adapting the writing parameters (SS, LP) to the material (Figure S9, Supporting Information). As in the case of Photoresist 1, the array fabricated with Photoresist 2 also gave rise to a full range of colors. The recorded λ_max_ values for this material showed the same trend of decreasing reflected wavelengths for increasing slicing and hatching distances. As expected, an overall blueshift for the range of reflected wavelengths and their corresponding inter‐layer distances was observed based on the relationship defined in Equation (2) (Tables S13–S15, Supporting Information). This effect can be expected when nanoparticles of smaller diameters are employed, as this results in decreased inter‐particle distances when the polymer microstructure experiences comparable levels of structural collapse, post‐development. The use of smaller nanoparticles also exacerbates the effect of DLW fabrication parameters on color generation, with even wider gamut structural color achievable (Δλ_max_ = 259 nm for Photoresist 1 versus Δλ_max_ = 322 nm for Photoresist 2 for HD = SL = 0.1 and 0.5 µm), noting that a higher laser power (100%) is necessary for fabrication in Photoresist 2.

To further explore how the effect of DLW could be complemented by self‐assembly, a third photoresist, consisting of the same nanoparticles and monomer composition as Photoresist 1, but with an increased nanoparticle concentration of 50 wt.% PNP1, was exploited in creating micropillar arrays. The recorded reflected wavelengths for the same micropillar array design were found to be very similar to those recorded for Photoresist 1, with a slight overall redshift (Tables S16–S18, Supporting Information). Although a higher nanoparticle concentration would be expected to cause a blueshift, the impact of the fabrication parameters (LP, SS) must also be taken into consideration. The latter had to be set considerably higher (90% LP, 10000 µm s^−1^) to obtain optimal polymerization of the 5 × 5 micropillar array compared to Photoresist 1 (60%, 10000 µm s^−1^). Such an increase in writing parameters will cause a different degree of polymerization, rendering direct comparison impractical. A lower concentration equivalent of Photoresist 1, at 40 wt.% PNP1, was also investigated. Micropillars fabricated with the 40 wt.% PNP1 photoresist under optimum fabrication conditions (100% LP, 5000 µm s^−1^), did not exhibit a visible color upon hydration, except for the microstructures produced with the largest hatching and slicing distance (SL, HD >0.4 µm), which resulted in the most collapsed structures (smallest inter‐particle distance). An optical image of the pillar array and representative scattering spectra for HD = 0.2 µm and HD = 0.5 µm, over the range of SL (0.1–0.5 µm), are shown in Figure S15 (Supporting Information).

**Figure 3:**
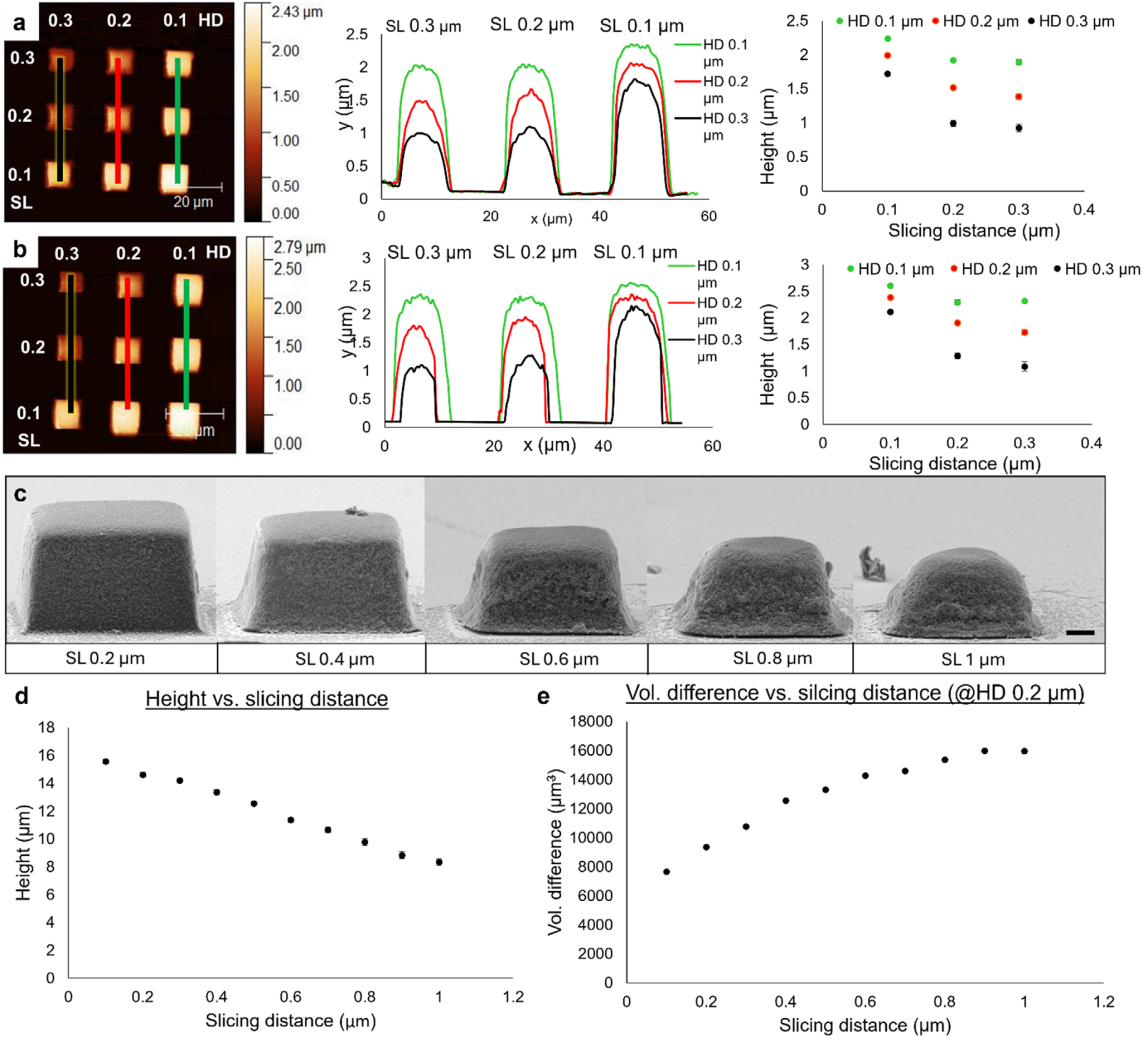
Structure characterisation. a,b) AFM topography image for a 3 × 3 cube (10 × 10 × 3 µm) array varying SL and HD from 0.1 to 0.3 µm, fabricated with Photoresist 1 at 60% LP, 10000 µm s^−1^, along with the corresponding height profile and height change with varying design parameters, respectively, in a) air and b) deionised water. Error bars represent the standard deviation for the measurements. Source data is provided in the ESI. c) SEM images taken at 80° tilt angle of 30 × 30 × 20 µm cube structures fabricated with Photoresist 3 (70% LP, 10000 µm s^−1^) displaying SL 0.2–1 µm at HD = 0.2 µm (scale bar represents 2 µm). d) Corresponding height values measured via SEM vs. slicing distances (Table S23, Supporting Information). e) Volume percentage difference between the cube design volume and post‐development cube volume against slicing distances.

To further investigate the influence of changing the HD and SL on the resulting microstructures, AFM topography measurements were performed in both dry and hydrated states. For this purpose, 3 × 3 micro‐cube arrays of design height = 3 µm, base = 10 × 10 µm, were fabricated using Photoresist 1, where SL and HD were increased from 0.1 µm to 0.3 µm in 0.1 µm increments (60% LP, 10000 µm s^−1^). AFM measurements after fabrication and post‐treatment (**Figure** **3**) indicate that increasing SL and HD both result in a contraction of structure height, as summarized in Tables S19 and S20 (Supporting Information). All structures are considerably contracted from the design height. Structures fabricated using SL = HD = 0.1 µm were contracted to 56% of the design height after fabrication. As in the case of optical characterization, holding one of the design parameters constant and varying the other led to only small variations in resulting heights. Varying both parameters, where SL = HD = 0.1, 0.2, 0.3 µm, enhanced this effect, resulting in height contractions (from design) of 44%, 62%, and 77%, respectively. Upon hydration, all structures were seen to increase in height (from 16% to 30%). As expected, combinations of small SL and HD values resulted in minimised swelling. Increasing values of either (or both) can be used to garner marginal increases in hydration.

While AFM gave useful information on the behavior of smaller nanocomposite structures, we further strived to understand the contraction of structures in the size regime used for optical characterisation, and the fidelity of structures resulting from larger SL and HD combinations. This can be further investigated using tilt SEM to explore structures after fabrication, as shown in Figure 3c. Using Photoresist 3, a series of microstructures with design dimensions of 30 × 30 × 20 µm were fabricated at 70%, 10000 µm s^−1^ with varied SL (SL 0.2–1 µm) and a moderate HD of 0.2 µm. A significant height difference was observed from SL = 0.1 µm (height = 15.6 ± 0.1 µm, n = 5) to SL = 1 µm (height = 8.3 ± 0.2 µm, n = 5) (Table S22, Supporting Information). To achieve highresolution fabrication, combined with wide‐gamut structural color, as outlined in subsequent sections, no values greater than HD = SL = 0.5 µm were used. Values above these are seen to significantly compromise the fidelity of the structures.

**Figure 4:**
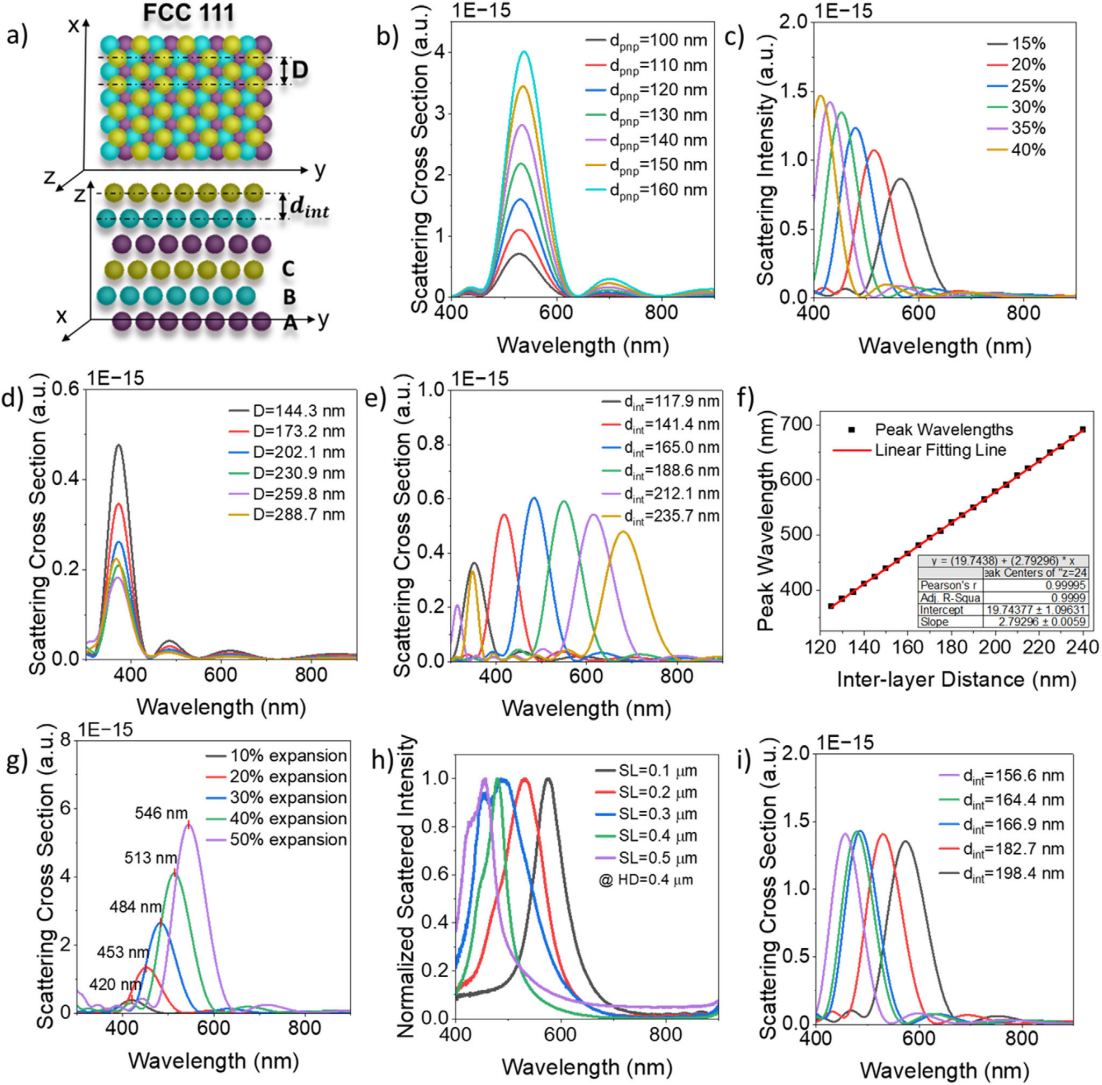
FDTD simulation results. a) Schematic diagrams representing the FCC (111) structure in the xy plane and yz plane. d_int_ is the inter‐layer distance in the FCC (111) plane in the z‐direction, and D denotes the closest inter‐particle distance in an FCC structure. FDTD simulation results of scattering cross‐section of an FCC (111) structure for b) varying particle diameter (d_PNP_ = 100–160 nm, D = 185 nm, d_int_ = 183 nm, nanoparticle RI = 1.49, host RI RI_bg_ = 1.46). c) Scattering cross‐section of an FCC (111) structure incorporating with different nanoparticle volume fractions (RI = 1.49, RI_bg_ = 1.46). d) Scattering cross‐section of a stretched FCC (111) with increasing inter‐particle distance in the xy plane (D = 144.3–288.7 nm, d_PNP_ = 117 nm, d_int_ = 125.2 nm, RI = 1.49, RI_bg_ = 1.474). e) Scattering cross‐section of a stretched FCC (111) structure with increasing inter‐layer distance in the z‐direction (d_int_ = 117.9‐235.7 nm, D = 153.4 nm, d_PNP_ = 117 nm, RI = 1.49, RI_bg_ = 1.474). f) FDTD simulated peak wavelength as a function of inter‐layer distance (d_int_ = 125‐240 nm, D = 185 nm, d_PNP_ = 117 nm, RI=1.49, RI_bg_ = 1.46). g) Scattering cross‐section of an expanded FCC (111) structure (30 vol% nanoparticles) with increasing expansion ratio in water (d_PNP_ = 117 nm, RI = 1.49). The corresponding D, d_int_, and RI_bg_ are listed in Table S25 (Supporting Information). The RI_bg_ values are estimated using the Maxwell‐Garnett approximation; h) Normalized experimental scattering spectra HD = 0.4 µm in the second row of Figure 2b. i) The simulated scattering cross section spectra using the inter‐layer distances (d_int_) calculated from the experimental spectra.

To further investigate the variation of slicing (SL) and hatching distances (HD), and the effect of their contribution on the spectra observed in Figure 2b, finite difference time domain (FDTD) (Lumerical) simulations were undertaken. This proved particularly useful, as direct cross‐sectional analysis of the polymer‐in‐polymer arrays using techniques such as Focused Ion Beam (FIB) or microtome proved ultimately unsuccessful because of the soft nature of the organic composite material. Variations of composite refractive index, PNP diameter, volume fraction, inter‐particle distance (x‐y and z direction), as well as structure swelling during hydration, were all considered. An FCC (111) assembly, shown in **Figure** **4**a, was modelled to simulate 3D ordering within the nanocomposite colloidal crystal. In this regard, it was necessary to define two untethered spacing variables: d_int_, which is the inter‐layer distance in the FCC (111) plane in the z direction, and D, which is the inter‐particle distance within a plane of the FCC structure. This enables the simulation of an augmented FCC (111) array wherein self‐assembly, followed by DLW, offers the potential to modulate spacing in x‐y and z directions independently, in a manner that is not possible using self‐assembly alone. This has many fascinating implications for the optical properties of the resulting structures.

Holding other geometrical parameters (such as d_int_ and D) constant, variation of particle diameter is seen to have a negligible effect on peak wavelength, as shown in Figure 4b. For traditional colloidal crystal assemblies, this would be a particularly contrived state, as particle diameter is inextricably linked to inter‐particle distances. This is not the case when combined with DLW, where particle size and spacing in all dimensions can be modulated. In this regard, it is enlightening to see that variation of particle size (particularly onerous to achieve synthetically) contributes very little to the ability to tune across the visible spectrum. As expected, the volume fraction of nanoparticles is seen to have a significant effect on scattering spectra. For d_PNP_ = 117 nm, the simulated spectra observed in Figure 4c show a gradual blueshift with increasing nanoparticle volume fraction, due to a decrease in FCC (111) inter‐layer distance (d_int_). It must be noted that such a range is practically rarely achievable, owing to inhibition of self‐assembly at low and high vol%. In addition, threshold volume (governed by Equation 1), is seen to increase with increasing nanoparticle size.^[^ ^15^ ^]^ The influence of the refractive index of PNP and that of the polymer matrix on the resulting spectra was found to be minimal (λ_max_ variation of 1–2 nm) within a refractive index variation range of 0.1, as shown in Figure S17 (Supporting Information). The refractive index contrast between the matrix polymer and the embedded nanoparticles is a critical factor influencing the intensity of the reflection band, as illustrated in Figure S17 (Supporting Information). This contrast becomes more pronounced in the hydrated sample, where water absorption lowers the refractive index of the polymer matrix, thereby enhancing the rdifference with the nanoparticles and resulting in a more intense reflection band.

To understand the effect of augmenting a photonic crystal in 3 dimensions, we probed the variation of spacing in all directions, independently. The simulated spectra in Figure 4d show that variation of interparticle distance in the xy plane has little bearing on the stop‐band of the resulting structure, affecting only the intensity of the resulting peaks. However, modulation of interlayer distance in the z‐direction is seen to be the dominant effect, as shown in Figure 4e (variation in d_int_) and Figure 4g (variation in structure expansion). The FDTD simulated scattering peak wavelengths (λ_max_) of an augmented FCC (111) structure show a linear relationship with increasing inter‐layer distance (d_int_)_,_ which can be linearly fitted by the equation λ_max_ = 19.744 + 2.793d_int_, as shown in Figure 4f. Using the peak wavelengths recorded for the second row of pillars (HD = 0.4 µm) in the micropillar array displayed in Figure 2b, the d_int_ values were calculated and employed to simulate the corresponding FDTD scattering spectra. These were found to nearly perfectly coincide with the experimental spectra as shown in Figure 4h,i. Similarly, the simulated d_int_ values (198.4, 182.7, 166.9, 164.4, 156.6 nm) showed a good match with the corresponding calculated d_int_ by Equation (2) (Table S9, Supporting Information). The main influence on the microstructural color, spanning the blue to red regions (Figure 2b), is the FCC (111) inter‐layer distance (d_int_).

**Figure 5:**
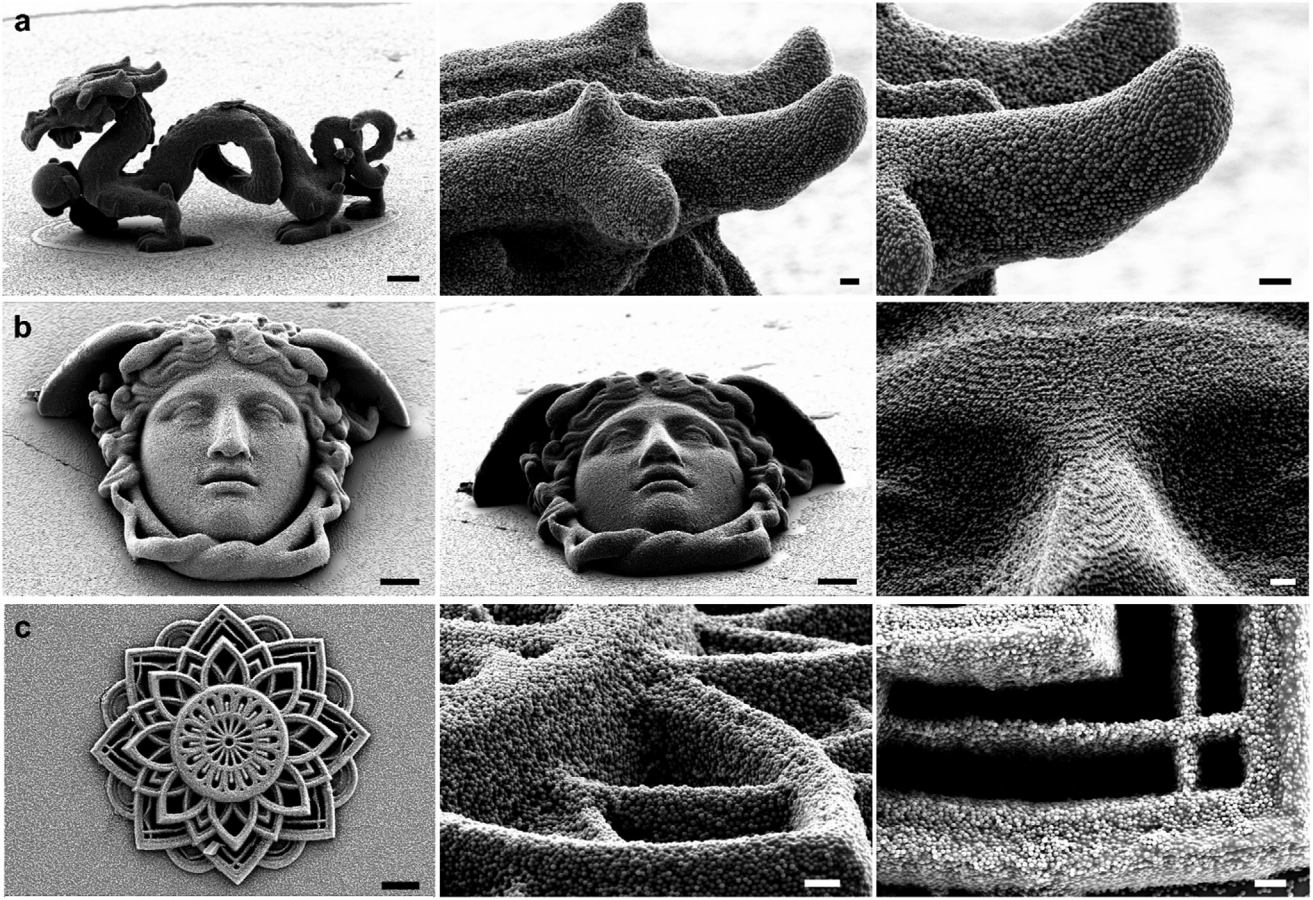
SEM characterization. a) SEM image of a dragon structure (left) highlighting the suspended middle part of the dragon's body (scale bar is 10 µm) and head detail (middle, right), highlighting particle assembly (scale bar is 1 µm). b) Head of a Medusa Rondanini structure (scale bar is 10 µm) and face detail (scale bar is 1 µm). c) Mandala structure (scale bar is 10 µm), with side‐view and top‐view detail (scale bar is 1 µm). All structures were fabricated in Photoresist 3 using optimised fabrication parameters; dragon structure (100% LP, 8000 µm s^−1^); Medusa Rondanini (100% LP, 9000 µm s^−1^); Mandala (100% LP, 7000 µm s^−1^).

The composite photoresists were also proven to render intricate structures with outstanding feature integrity. As observed from **Figure** **5**a, Photoresist 3 yielded structures with stable overhang features, denoted by the body, tail, head, and pearl of the dragon microstructure. Other than the dragon model, the Rondanini Medusa microstructure (Figure 2b) was also used to exemplify a complex structure characterized by smooth, curved, entwined, and arched features, which proved to be a successful feat. It was also possible to obtain preservation of complex details, as highlighted by the mandala designs (Figure 5c; Figures S18 and S19, Supporting Information), with the smallest features in XY found to be ≈211 ± 37 nm in width (Figure S19, Supporting Information). Additionally, height elements for the Mandala design revealed successful reproduction of Z features with step heights of 3.7 ± 0.06 µm and 1.9 µm ± 0.05 (Figure 5c; Figure S18, Supporting Information). Pyramid structures (24 µm in height) were also fabricated, as their design allows for easy visualisation of PNP self‐assembly, as well as exemplifying the realisation of 3D structures that come to a tip of less than 1 µm in width (Figure S20, Supporting Information). Similar results were obtained with all of the other photoresists. As such, hollow structures of 40 µm in height and having a designed wall thickness of 2 µm were also successfully achieved using Photoresist 1 (Figure S21, Supporting Information).

**Figure 6:**
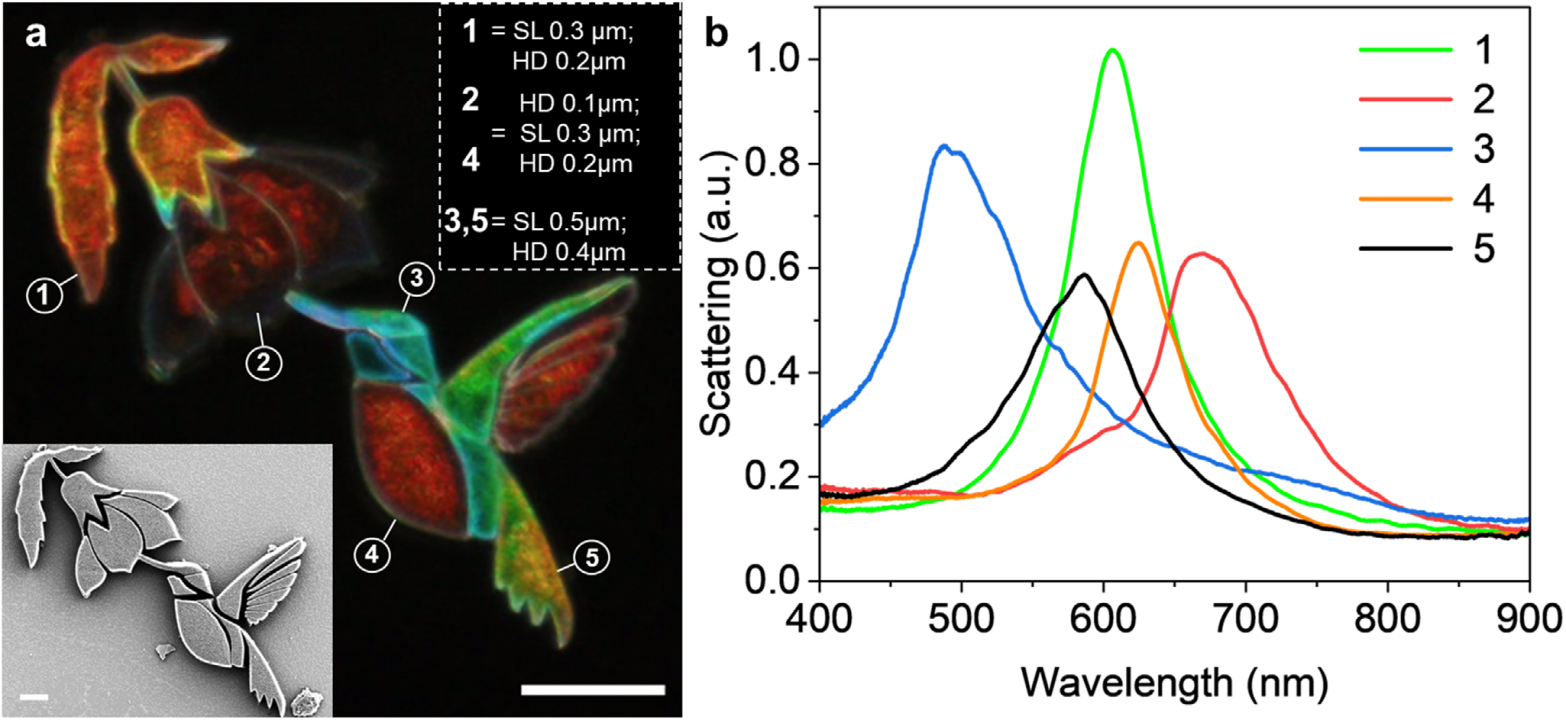
Art at the microscale. a) Dark field microscopy image of a structural color picture showing a hummingbird extracting nectar from a flower, with SEM picture insert (scale bar = 20 µm), fabricated using Photoresist 3, where each color was achieved by modulating fabrication parameters (region 1: SL = 0.3 µm, HD = 0.2 µm, SS = 9000 µm s^−1^, 100% LP; region 2 & 4: SL = 0.3 µm, HD = 0.1 µm and HD = 0.2 µm respectively, SS = 7000 µm s^−1^, 100% LP; region 3 & 5: SL = 0.5 µm, HD = 0.4 µm, SS = 4500 µm s^−1^, 100% LP). The scale bar represents 50 µm (Design height 20 µm). b) Corresponding scattering spectra for color sections of the microstructure.

As proof of concept, different slicing and hatching distances were employed to realize a vibrant multi‐color microstructure representing a hummingbird feeding on a flower's nectar (**Figure** **6**; Figure S22, Supporting Information), by employing a single photoresist material (Photoresist 3) and within a single fabrication step. This was achieved by sketching a digital picture and converting each of the differently colored sections into STL files. These files could then be imported into the software used for DLW (DeScribe) where each hatching and slicing parameter can be set. Each of the regions could then be fabricated in a programmed order, which also allowed for LP variation for each region. The different parameters gave rise to very distinct colors with well‐defined bands in the corresponding recorded scattering spectra (Figure 6b).

This work is further expanded to encompass a responsive polymer matrix, based on a phenyl boronic acid (PBA) monomer (Photoresist 4, Table S5, Supporting Information), containing 55 wt.% of PNP (d_SEM_ = 173±7 nm; d_hyd_ = 241±5 nm). Phenyl boronic acids are widely known for their sugar‐sensitive properties and have been extensively exploited for sugar sensing applications.^[^ ^34^ ^]^ At pH values below the pK_a_ of PBA (pK_a_ ≅ 8.2–8.6)^[^ ^35^ ^]^ PBA is found in its neutral trigonal planar form. With the introduction of diol‐containing species, such as fructose, the PBA binds to the syn‐periplanar hydroxyl groups available on the 5‐membered ring of the β form of D‐fructose to give a boronate ester, where the boron is negatively charged (Figure S23, Supporting Information).^[^ ^34^ ^]^ When included in a hydrogel polymer network, this binding event causes hydrogel expansion. Introduction of fructose (100 mM) was used to vary the periodicity of the ordered nanocomposite, thereby further changing the reflected color, to produce a photonic chemical sensor (Figure S24, Supporting Information). The fabricated microcubes exhibited a color change when changing their environment from PBS to fructose (100 mm), with λ_max_ increases of 35 nm, from 565 nm (PBS) to 600 nm (100 mm fructose in PBS), when a SL = 0.3 µm and HD = 0.4 µm were employed. The λ_max_ increase upon fructose addition, i.e., bathochromic shift, is dependent on the fabrication and design parameters, i.e., SL and HD, thereby allowing for further tuning of the response (i.e. λ_max_ range).

Successful DLW fabrication of tuneable structurally colored microstructures by employing self‐assembled polymer nanoparticles was realised herein for the first time. This was achieved using PNPs of two different sizes (PNP1 – 117 ± 12 nm, PNP2 – 100 ± 7 nm), synthesized via emulsion‐free polymerization. Incorporation of either nanoparticle species at weight concentrations of 45 or 50 wt.% gave rise to structurally colored photoresists, which were used for DLW to create complex high‐resolution microstructures with precise control over interparticle distance and structural color. The experimental evidence demonstrates how the color change can be directly programmed by fine‐tuning design and fabrication parameters. The simulations confirm that this is achieved by variation of interparticle distance. Application of this technology in stimuli‐responsive polymers will enable the development of dynamic micro‐sensors.

## 3 Conclusion

The work presented herein represents the first report of achieving a full gamut of bright structural colors by employing a single polymer nanoparticle‐composite material. This achievement is possible through the tuning of the interparticle distance via variations of fabrication parameters, such as slicing and hatching distance. The findings demonstrated above not only prove the efficiency of this novel approach to obtain bright and precisely controlled structural color, but also show a new fabrication route that promises great versatility in terms of materials and applications. This approach promises a broadly applicable method for the realisation of micrometer‐size pixels of tunable structural color from self‐assembled colloidal particles. To our knowledge, the fabrication and characteristics of the polymer composite structures presented herein are the first of their kind, showing unprecedented potential for swellable soft hydrogel structures with brilliant and programmable colors in water, all of which were achieved through a one‐step method. These structures will undoubtedly open the possibility to new applications in fields such as chemical encryption, biomedical engineering, and analyte sensing.

## 4 Experimental Section

### Materials

Phenylbis(2,4,6‐trimethylbenzoyl) phosphine oxide (PBPO), 3‐(trimethoxysilyl) propyl methacrylate 98%, methyl methacrylate (MMA), hydroxyethyl acrylate (HEA), 1,6–Hexanediol diacrylate (HDDA), triglycerol diacrylate (TGDA), hydroxyethyl acrylamide (HEAA), 3‐acrylamidophenylboronic acid (98%) (PBA), potassium persulfate (PPS), and acetic acid analytical grade were obtained from Sigma–Aldrich, Ireland, and used as received. SR9035 (ethoxylated (15) trimethylolpropane triacrylate) was purchased from Sartomer Arkema and used as received. All solvents, including methanol, ethanol, acetone, isopropyl alcohol (IPA), were high‐performance liquid chromatography grade (HPLC) purchased in anhydrous form, from Sigma–Aldrich and used without further purification.

### Emulsion Polymerization

Two different‐sized poly(methyl methacrylate‐co‐1,6‐hexanediol diacrylate‐co‐hydroxy ethyl acrylate) nanoparticles, PNP1 and PNP2, were synthesized via emulsion polymerization in deionised (DI) water, using potassium persulfate as an initiator, methyl methacrylate (MMA) as the main monomer, hydroxyethyl acrylate (HEA) as a hydrophilic comonomer (11 and 11.8 mol% MMA respectively), and 1,6‐Hexanediol diacrylate (HDDA) as a crosslinker (5.7 mol% to MMA), as detailed in Table S1 (Supporting Information).

The monomers were added to a boiling solution of NaCl in DI water at 120 °C, stirring at 1010 rpm. After 20 min, the initiator (PPS) was added, and the temperature was brought down to a constant 80 °C for 3 h. The particles were dialysed in DI water for 48 h and subsequently mixed with ion exchange resin. Afterward, they were washed and isolated via repeated centrifugation in DI water and ethanol.

The obtained nanoparticles were then analysed by SEM and DLS. The SEM measurements showed an average particle diameter for PNP1 of 117 ± 12 nm, and for PNP2 of 100 ± 7 nm (Figures S1 and S2, Supporting Information). DLS measurements in DI water showed an average hydrodynamic diameter of d_hyd_ = 186 ± 3 nm, PDI = 0.077 for PNP1, and d_hyd_ = 160 ± 2 nm, PDI = 0.02 for PNP2 (Figure S2, Supporting Information).

### Photoresist Preparation

Photoresists 1 and 3 (Tables S2 and S4, Supporting Information), were composed of 45 and 50 wt.% PNP1, respectively, together with ethoxylated (15) trimethylolpropane triacrylate (SR 9035) and the photoinitiator, phenylbis(2,4,6 trimethyl benzoyl) phosphine oxide (PBPO) (Tables S2 and S4, Supporting Information). Photoresist 2 (Table S3, Supporting Information) was composed of 45 wt.% PNP2, as well as the same chemical reagents as Photoresists 1 and 3. A sugar‐responsive nanocomposite photoresist, based on a phenyl boronic acid (PBA) monomer (Table S5, Supporting Information) containing 55 wt.% of PNP (d_SEM_ = 173 ± 7 nm; d_hyd_ = 241 ± 5 nm), was prepared in the same manner according to the composition described in Table S5 (Supporting Information).

The monomers were mixed with the respective PNPs using an overhead IKA stirrer at 350 rpm for several hours. The mixtures were subsequently heated at 60 °C to ensure solvent evaporation, after which the photoinitiator was added to the photoresist and dissolved via thorough sonication. At least 24 h ahead of fabrication, a drop of unpolymerized photoresist was deposited into a capillary cell comprising a fluorinated ethylene propylene (FEP) sheet (top), and a silanized high precision glass coverslip (170 ± 5 µm; 30 mm in diameter) (bottom) (Thermofisher Scientific), separated by 2 strips of pressure‐sensitive adhesive (PSA). The prepared slide was then stored in the dark at 7 °C (Figure S3, Supporting Information). This deposition method enabled the capillary‐driven self‐assembly of the PNP within the photoresist, resulting in structural color being observed (Figure S4, Supporting Information).

### Direct Laser Writing Fabrication

Two‐photon polymerization was induced by a focused laser beam from a 780 nm femtosecond laser in a commercial DLW workstation (Photonic Professional, Nanoscribe GmbH). Fabrication of the 3D soft structures was performed in an oil‐immersion configuration using a 63x immersion objective (NA = 1.4, WD = 190 µm) (Zeiss, Plan Apochromat). For this, a drop of Zeiss Immersol oil was placed in the center of the home‐made capillary cell containing the self‐assembled photoresist (Figure S3, Supporting Information) and brought in contact with the 63x immersion objective for microfabrication.

The laser power and the scan speed for fabrication of the 3D structures were set between 60–100% LP (30–50 mW), and scan speeds varied between 5000 and 10000 µm s^−1^, without the use of any contours. After fabrication, all microstructures were developed in a 30:70 DI water:isopropyl alcohol (IPA) mixture for a minimum of 5 min at a temperature of 65 °C, after removing the top FEP film. Successful fabrication was confirmed using optical microscopy.

To enhance the adhesion of the acrylic‐based microstructures to the glass substrate, the glass slides were cleaned with acetone, isopropanol, ethanol, methanol, and DI water, dried, and exposed to oxygen plasma for 2 min. Following this, the slides were functionalized with 3‐(trimethoxysilyl) propyl methacrylate (3 vol% in EtOH with 0.1 vol% acetic acid) for 1 h, rinsed in EtOH, and dried with nitrogen.

### Microscopy and Dark Field Measurements

Dark field imaging was performed on an Olympus BX53 microscope using a 20X objective lens (NA = 0.4, Olympus LMPlanFL N). The images and spectra were taken using a CCD camera and an Andor 230i spectrometer, respectively. The normalized scattering spectra were obtained using (SS_sample_‐SS_bg_)/(SS_diffuser_‐SS_bg_), where SS_s__ample_ is the scattering spectrum measured from the sample, SS_bg_ is the background spectrum from the ambient environment, and SS_diffuser_ is the reference spectrum measured using a glass cell filled with solvents placed on a ceramic diffuser.

### Scanning Electron Microscopy

Scanning electron microscopy of the PNP and 2PP‐fabricated microstructures was carried out using a Zeiss ULTRA plus scanning electron microscope. An accelerating voltage of 5 kV was used under SE2 mode to acquire all images. Prior to SEM imaging, the structures were coated with ≈15 nm Au/Pd using a Cressington sputter coater 208HR. A 57 × 0.1 mm Au/Pd target (TED PELLA INC.) was used to coat the structures under an inert atmosphere of argon for 15 s. The average particle diameter was measured from top‐down SEM images using the particle analysis function on ImageJ. A minimum of 25 diameter measurements were obtained for each PNP, to ensure a representative sample of the particles. The SEM height and width measurements for the microcube arrays fabricated with Photoresist 3 (Figure 3c,d; Figure S16, Tables S22 and S23, Supporting Information) were obtained at an 80° angle, measured with ImageJ, considering the average of 5 measurements for each structure. The same approach was applied for the mandala feature size measurement, based on SEM images taken top‐down (Figure S19, Supporting Information).

### Atomic Force Microscopy

Atomic force microscopy of microcubes (10 µm x 10 µm x 3 µm = l x w x h) fabricated via 2PP using Photoresist 1 was carried out. The samples were imaged in air and in DI water, using an MFP‐3D AFM system (Asylum Research). A Pt/Ir‐coated conductive tip (PPP‐EFM Nanosensor, with tip radius <25 nm, resonant frequency of ≈350 kHz, and a spring constant of 40 N m^−1^ was used. AFM images were obtained using contact mode with 256 lines per scan direction and with a scan rate of 0.4 Hz and a scan angle of 90°. The AFM height images were processed using Gwyddion AFM software (version 2.6).

### Dynamic Light Scattering

DLS measurements were performed on a Malvern Zetasizer Nano ZS, using a 633 nm HeNe laser. Samples were prepared by diluting the PNP dispersions in deionised water. Samples were tested in quartz cuvettes with a path length of 1 cm. The machine was operated in a backscatter mode at an angle of 173°. Samples were equilibrated for 120 s at 25 °C prior to measurement. Parameters at 25 °C of media refractive index n = 1.334 and media viscosity of 0.890 and sample refractive index n_p‐MMA_ = 1.4905 were assumed in the measurement.

### Numerical Simulations

The finite difference time domain (FDTD) solver in Lumerical software was used to simulate the scattering cross section of the structures. The FCC (111) structure model is shown in Figure 4a. The structures were surrounded by the TFSF source and 6 DFT monitors. And the PML boundary conditions were applied in all 3 directions. The monitor in the reflection position was used to capture the reflected signal. The structure region was meshed with a 10 nm × 10 nm × 10 nm resolution. The refractive index of the PMMA nanoparticle was set at 1.49.^[^ ^31^ ^]^

## Conflict of Interest

The authors declare no conflict of interest.

## Author Contributions

T.F. and J.Q contributed equally to this work. T.F. and J.Q. should be considered joint first authors. T.F. conducted sample preparation, DLS, DLW, and data analysis. J.Q. performed spectroscopic measurements, FDTD simulations, and analysis. S.K. performed and analysed AFM measurements. T.F., L.F., and C.D. wrote and revised the manuscript. A.L.B. and J.Q. contributed to writing and editing. All authors contributed to the discussion and interpretation. L.F. and C.D. supervised and coordinated the project.

## Supporting information



Supporting Information

## Data Availability

The authors declare that all data supporting the findings of this study are available within the article and its Supplementary Information or from the corresponding author upon reasonable request.
