# Supplementary material for: Direct Laser Writing of Polymer Nanocomposites for Tunable Structural Color
Source: Adv Mater. 2025 Jul 9;37(43):2504116. doi: 10.1002/adma.202504116 (PMC12574619; doi:10.1002/adma.202504116)
Supplement: Supplementary file 1 — Supporting Information [file ADMA-37-2504116-s001.pdf]

ADVANCED MATERIALS

Supporting Information

for *Adv. Mater.*, DOI 10.1002/adma.202504116

Direct Laser Writing of Polymer Nanocomposites for Tunable Structural Color

Teodora Faraone, Jing Qian, Srikanth Kolagatla, A. Louise Bradley, Larisa Florea and Colm Delaney**

Direct Laser Writing of Polymer Nanocomposites for Tuneable Structural Color

Teodora Faraone,[1†] Jing Qian,[2†] Srikanth Kolagatla,[1] A. Louise Bradley,[2] Larisa Florea[1], Colm Delaney[1]**

† TF and JQ should be considered joint first author

[1]School of Chemistry & AMBER
The SFI Research Centre for Advanced Materials and BioEngineering Research
Trinity College Dublin
Dublin 2, Ireland
E-mail: CDELANE5@tcd.ie; FLOREAL@tcd.ie;

[2]School of Physics and AMBER
The SFI Research Centre for Advanced Materials and BioEngineering Research
Trinity College Dublin
Dublin 2, Ireland

Supplementary Information

Design Files: Several of the 3D designs used for the DLW fabrication of microstructures presented in this work, are available at thingiverse.com as detailed below:

Octopus by Kempi05, licensed under CC BY-SA.

https://www.thingiverse.com/thing:776710

Flexi Squid! by AGD_Toys, licensed under CC BY-SA.

https://www.thingiverse.com/thing:3576075

Halloween Pumpkin light by dennisheijmans, licensed under CC BY-SA.

https://www.thingiverse.com/thing:4614908

Harry Potter Sorting Hat by Volt81885, licensed under CC BY-SA.

https://www.thingiverse.com/thing:4338057

Ryujin by LennyFace, licensed under CC BY-SA.

https://www.thingiverse.com/thing:6503933

The Medusa Rondanini by CosmoWenman, licensed under CC BY-SA.

https://www.thingiverse.com/thing:196047

mandala-22 by green_long, licensed under CC BY-SA.

https://www.thingiverse.com/thing:5090091

mandala-12 by green_long, licensed under CC BY-SA.

https://www.thingiverse.com/thing:4744597

mandala-23 (L4) by green_long, licensed under CC BY-SA.

https://www.thingiverse.com/thing:5135408

*Emulsion Polymerization **PNP1**:* Methyl methacrylate (MMA) (3.276 g), 6-Hexanediol diacrylate (HDDA) (396.9 x10^{-3}g), and hydroxyethyl acrylate (HEA) (418.6 x10^{-3} g), were added to a boiling solution of deionised (DI) water (160 mL) and NaCl (40 x10^{-3} g) at 120 ºC stirred at 1010 rpm. After 20 minutes, the initiator PPS (95.8 x10^{-3} g in 4.5 mL of DI water) was carefully pipetted to the solution, after which the temperature was reduced to 80 ºC for 3 hours.

*Emulsion Polymerization **PNP2***: MMA (3.278 g), HDDA (397.0 x10^{-3} g), and HEA (418.5 x10^{-3} g), were added to a boiling solution of DI water (160 mL) and NaCl (40 x10^{-3} g) at 120 ºC at 1010 rpm. After 20 minutes, the initiator PPS (95.8 x10^{-3} g in 4.5 mL of DI water) was carefully pipetted to the solution, after which the temperature was reduced to 80 ºC for 3 hours.

The particles were dialysed against DI water for 48 hours and subsequently mixed in ion exchange resin. Afterwards, they were washed and isolated via repeated centrifugation in DI water and ethanol.

Supplementary Table S1. Reactants used for the synthesis of PNP1 and PNP2.

Reactant	MW (g/mol)	Density (g/mL)	Volume (mL)	Weight (g)	Mol (mmol)	Mol% (wrt MMA)
	**PNP1**					
**MMA**	100.121	0.936	3.5	3.276	32.72	100%
**HDDA**	226.27	1.01	0.393	0.397	1.75	5.36%
**HEA**	116.1	1.011	0.414	0.419	3.61	11%
	**PNP2**					
**MMA**	100.121	0.936	3.5	3.278	32.74	100%
**HDDA**	226.27	1.01	0.393	0.397	1.76	5.36%
**HEA**	116.1	1.011	0.414	0.419	3.60	11%

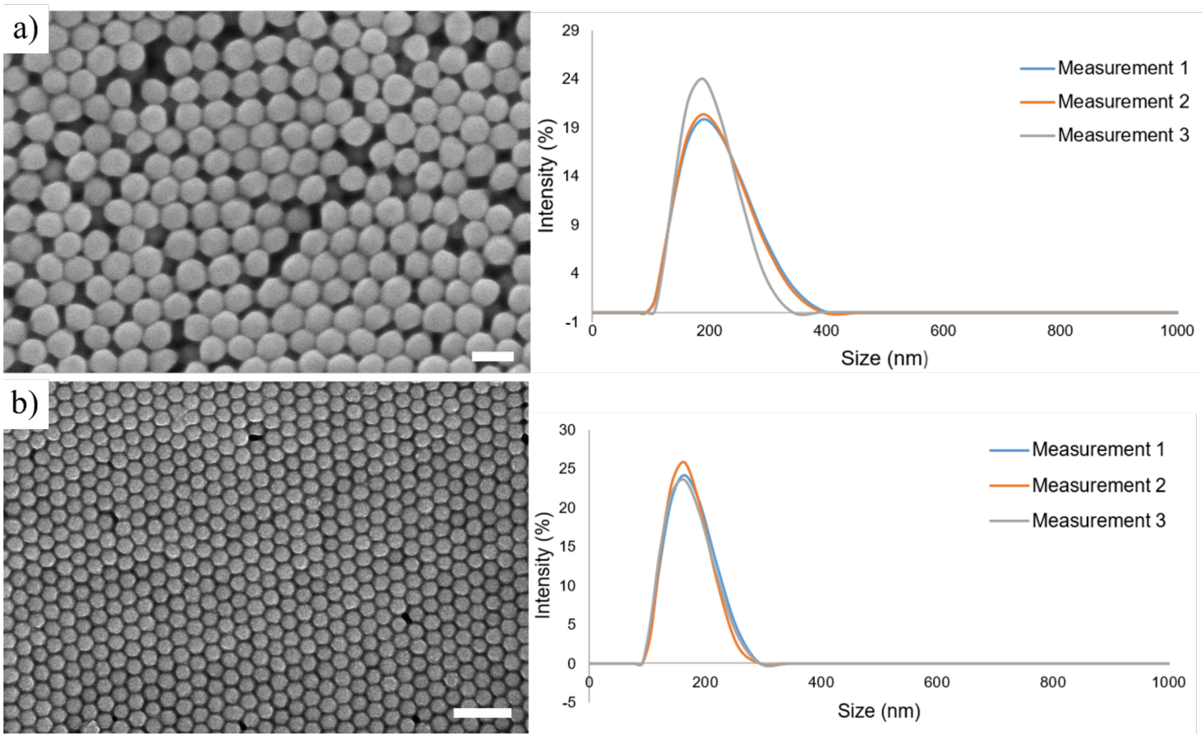

Supplementary Figure S1. SEM images (scale bars represent 200 nm) and hydrodynamic diameter DLS measurements corresponding to **a** PNP1 and **b** PNP2.

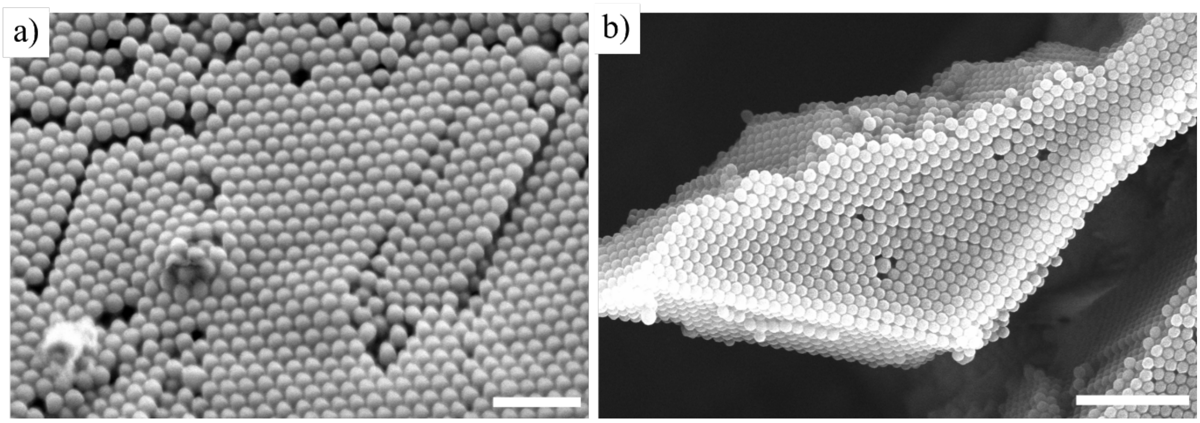

Supplementary Figure S2. SEM images of self-assembled films of **a** PNP1 and **b** PNP2 film (scale bars represent 1 μm).

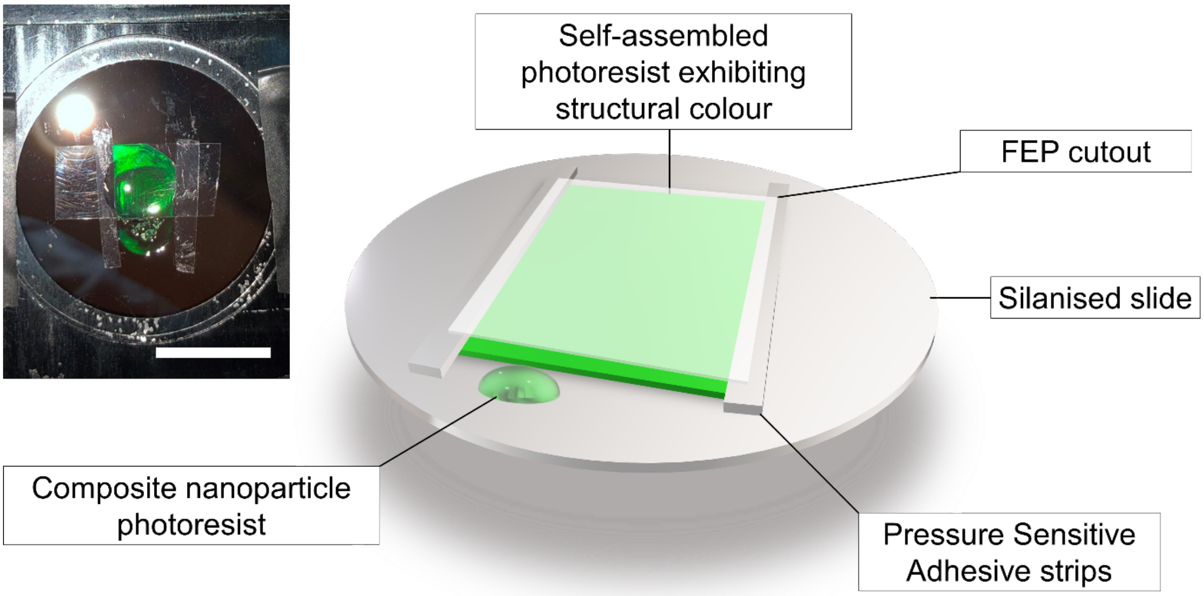

Supplementary Figure S3. Capillary cell for photoresist self-assembly and fabrication via DLW. The photoresist is deposited at the bottom of a capillary cell consisting of an FEP top slide and a silanized cover slip, separated by pressure-sensitive adhesive (PSA) spacers. Inset shows an image of the uncured Photoresist 1 in the cell pre-fabrication. Scale bar represents 1 cm.

Supplementary Table S2. Composition of Photoresist 1.

Reactant	MW (g/mol)	Weight (mg)	Mol (mmol)	Mol% (wrt SR 9035)	Weight %
**PNP1**	-	243	-	-	44.8
**SR 9035**	945	297	0.314	100	54.6
**PBPO**	418.46	3.3	8×10^{-3}	2.5	0.6

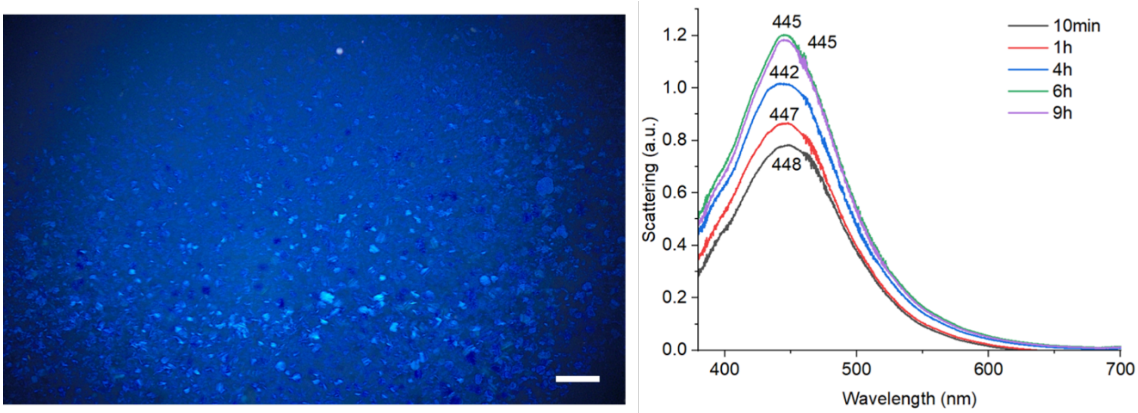

Supplementary Figure S4. Dark field optical microscope image of uncured 45 wt% PNP2 self-assembled film (24 hours after deposition) (Scale bar represents 200 µm). Scattering spectra monitoring self-assembly over several hours.

Supplementary Table S3. Composition of Photoresist 2.

Reactant	MW (g/mol)	Weight (mg)	Mol (mmol)	Mol% (wrt SR 9035)	Weight %
**PNP2**	-	120.8	-	-	44.99
**SR 9035**	945	146.4	0.154	100	54.53
**PBPO**	418.46	1.3	3.1×10^{-3}	2	0.48

Supplementary Table S4. Composition of Photoresist 3.

Reactant	MW (g/mol)	Weight (mg)	Mol (mmol)	Mol% (wrt SR 9035)	Weight %
**PNP1**	-	199	-	-	50.35
**SR 9035**	945	194.3	0.205	100	49.17
**PBPO**	418.46	1.9	4.5×10^{-3}	2.2	0.48

Supplementary Table S5. Composition of the sugar-responsive nanocomposite photoresist, based on a phenyl boronic acid (PBA) monomer containing 55 wt% of PNP (d_{SEM} = 173±7 nm; d_{hyd} = 241±5 nm). The quantities of each of the components N-Hydroxy ethyl acrylamide (HEAA), triglycerol diacrylate (TGDA), 3-acrylamidophenylboronic acid (PBA) and phenylbis(2,4,6 trimethyl benzoyl) phosphine oxide PBPO) are detailed below.

Reactant	MW (g/mol)	Density (g/mL)	Volume (µL)	Weight (mg)	Mol (mmol)	Mol% (wrt HEAA)	Weight %
**PNP**	-	-	-	180	-	-	54.9
**HEAA**	115.13	1.111	61.3	68.1	0.59	100	20.8
**TGDA**	348.35	1.237	47.8	59.1	0.17	28.7	18.0
**PBA**	190.99	-	-	19.2	0.10	17.0	4.8
**PBPO**	418.46	-	-	1.5	0.0036	0.6	0.46

Supplementary Table S6. Three polymerised SR9035 films and two polymerised 40 wt% PNP1 - SR9035 films were made, and the corresponding refractive indices measured by refractometer (Shanghai CSOIF CO., LTD). The refractive indices of both dry and hydrated films are summarized in the table below, where n represents the number of is the number of measurements.

Polymer Film Sample	SR9035		40 wt% PNP1 - SR9035 composite	
	Dry	Hydrated	Dry	Hydrated
**Refractive Index**	**1.4931±0.0011** (n = 7)	**1.4635±0.0020** (n = 6)	**1.4914±0.0020** (n = 8)	**1.4589±0.0016** (n = 5)

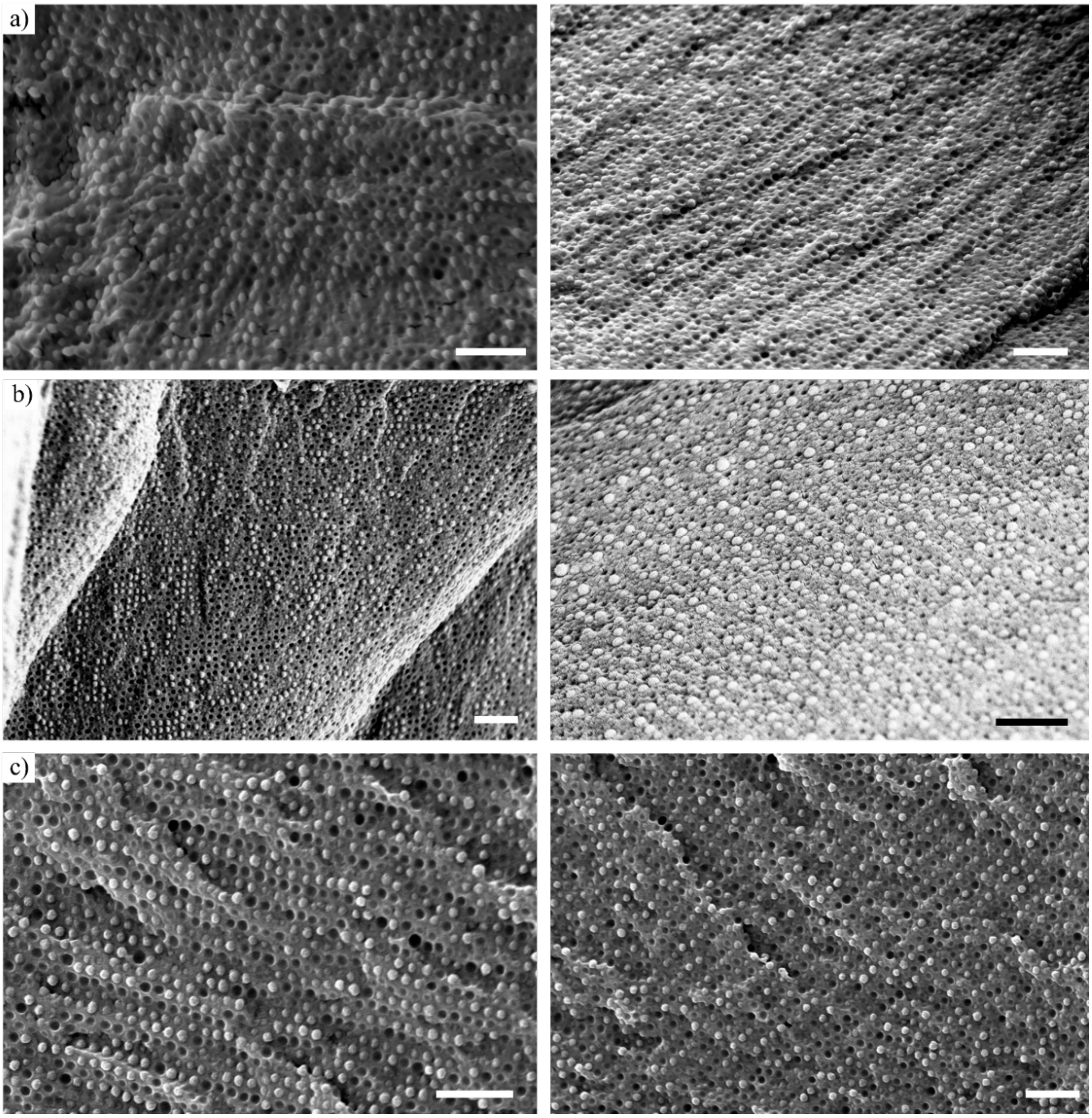

Supplementary Figure S4. SEM images of sheared nanocomposite polymer films showing self-assembled polymer nanoparticles in **a** Photoresist 1, **b** Photoresist 2, **c** Photoresist 3. (Scale bars represent 1 μm).

Supplementary Table S7. Details of hydrated micropillar array upon varying both slicing (SL) and hatching distance (HD) from 0.1 to 0.5 μm, fabricated in Photoresist 1 (60% LP, 10000 μm/s). λ_{max} values measured from scattering spectra, interparticle distances calculated using Eq. 2.

SL (μm)	HD (μm)	λ_{max} (nm)	d_{int} (nm)
0.1	0.1	678	232
0.2	0.2	567	194
0.3	0.3	513	176
0.4	0.4	479	164
0.5	0.5	419	143

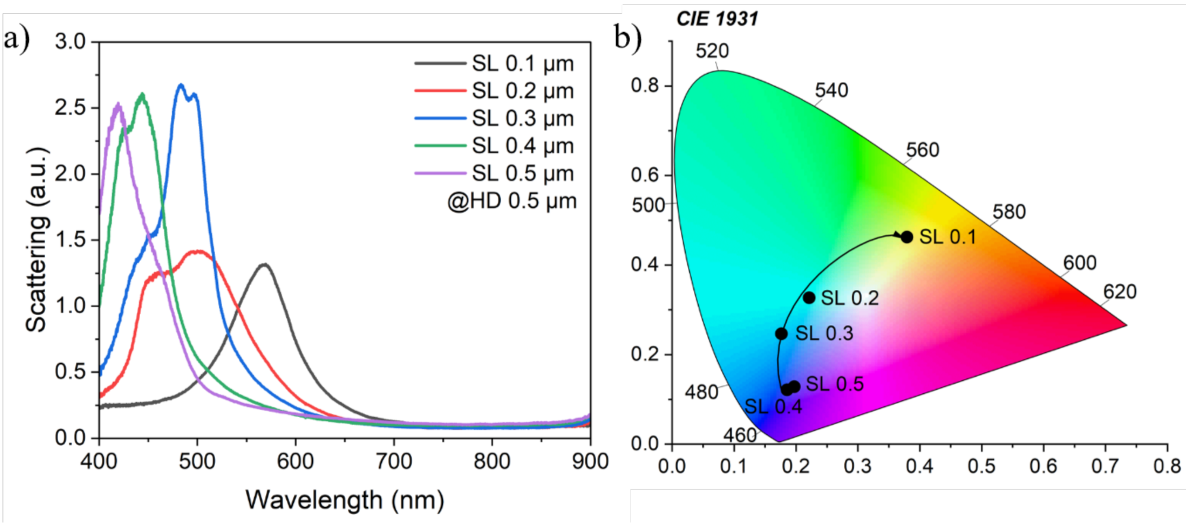

Supplementary Figure S5. a Scattering spectra of hydrated micropillar array fabricated in Photoresist 1, varying SL = 0.1 – 0.5 µm at constant HD = 0.5 µm (60% LP, 10000 µm/s) and **b** corresponding CIE diagram.

Supplementary Table S8. λ_{max} values measured upon varying SL = 0.1 – 0.5 µm at constant HD = 0.5 µm within Photoresist 1 micropillar array (60% LP, 10000 µm/s).

SL (µm)	HD (µm)	λ_{max} (nm)
0.1	0.5	569
0.2	0.5	500
0.3	0.5	483
0.4	0.5	444
0.5	0.5	419

Supplementary Table S9. λ_{max} values measured upon varying SL = 0.1 – 0.5 µm at constant HD = 0.4 µm in Photoresist 1 (60% LP, 10000 µm/s). λ_{max} values measured from scattering spectra, interparticle distances calculated using Eq. 2.

SL (µm)	HD (µm)	λ_{max} (nm)	d_{int} (nm)
0.1	0.4	574	197
0.2	0.4	530	182
0.3	0.4	486	166
0.4	0.4	479	164
0.5	0.4	457	156

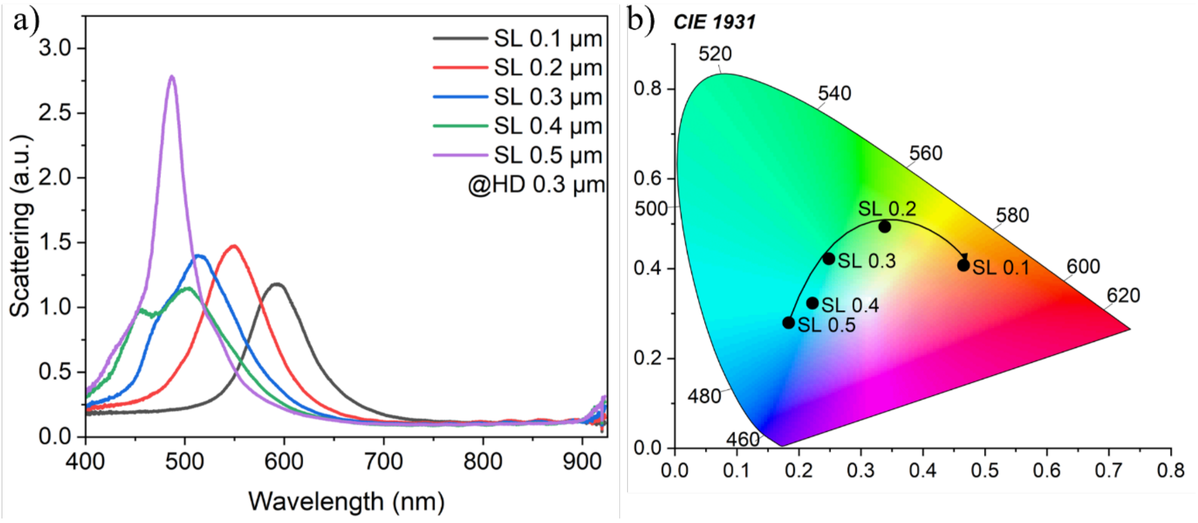

Supplementary Figure S6. a Scattering spectra of hydrated micropillar array fabricated in Photoresist 1, varying SL = 0.1 – 0.5 μm at constant HD = 0.3 μm (60% LP, 10000 μm/s) and **b** corresponding CIE diagram.

Supplementary Table S10. λ_{max} values measured upon varying SL = 0.1 – 0.5 μm at constant HD = 0.3 μm in Photoresist 1 (60% LP, 10000 μm/s).

**SL (μm)**	**HD (μm)**	**λ_{max} (nm)**
0.1	0.3	593
0.2	0.3	550
0.3	0.3	513
0.4	0.3	505
0.5	0.3	487

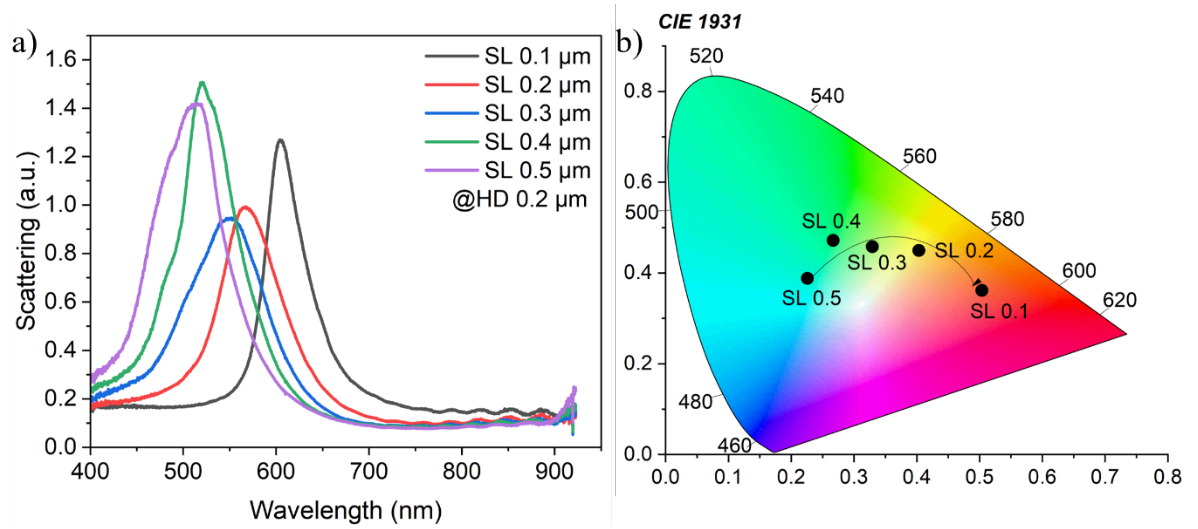

Supplementary Figure S7. a Scattering spectra of hydrated micropillar array fabricated in Photoresist 1, varying SL = 0.1 – 0.5 μm at constant HD = 0.2 μm (60% LP,10000 μm/s) and **b** corresponding CIE diagram.

Supplementary Table S11. λ_{max} values measured upon varying SL = 0.1 – 0.5 µm at constant HD = 0.2 µm in Photoresist 1 micropillar array (60% LP (30 mW), 10000 µm/s).

**SL (µm)**	**HD (µm)**	**λ_{max} (nm)**
0.1	0.2	604
0.2	0.2	567
0.3	0.2	552
0.4	0.2	520
0.5	0.2	515

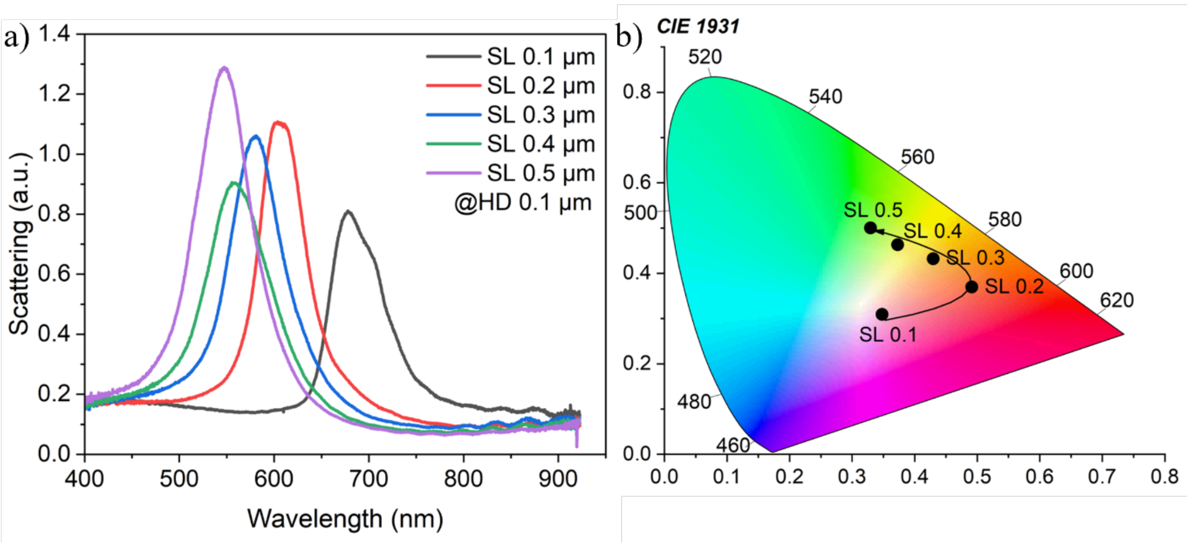

Supplementary Figure S8. a Scattering spectra of hydrated micropillar array fabricated in Photoresist 1, varying SL = 0.1 – 0.5 µm at constant HD = 0.1 µm (60% LP, 10000 µm/s) and **b** corresponding CIE diagram.

Supplementary Table S12. λ_{max} values measured upon varying SL = 0.1 – 0.5 µm at constant HD = 0.1 µm in Photoresist 1 micropillar array (60% LP, 10000 µm/s).

**SL (µm)**	**HD (µm)**	**λ_{max} (nm)**
0.1	0.1	678
0.2	0.1	604
0.3	0.1	581
0.4	0.1	555
0.5	0.1	547

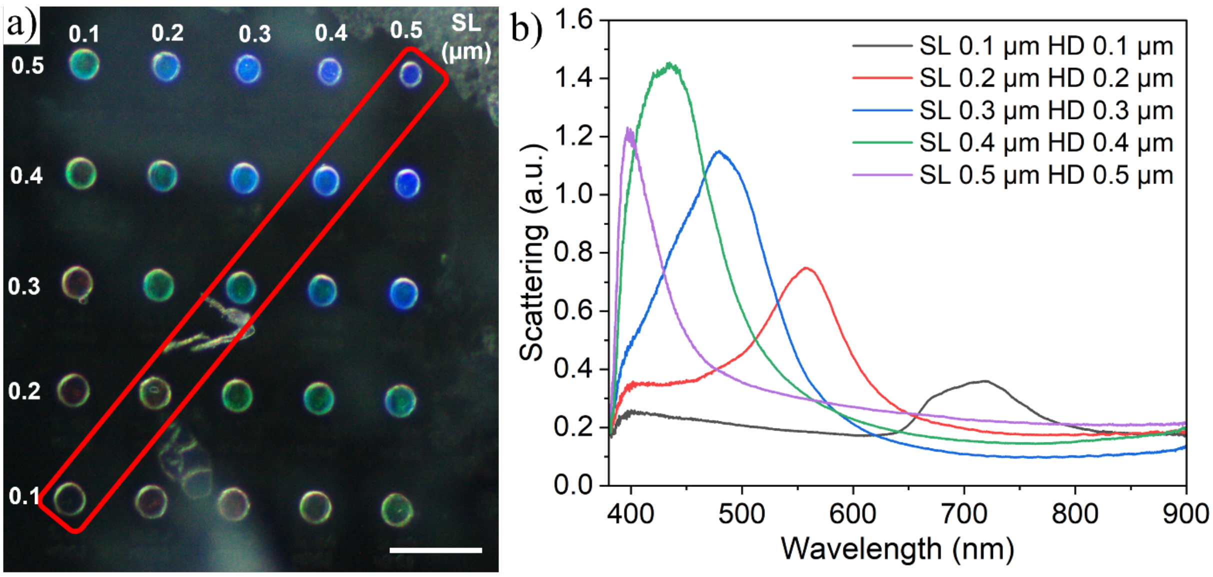

Supplementary Figure S9. a Dark field optical microscope image of 5 x 5 micropillar array, varying both slicing and hatching distance from 0.1 to 0.5 μm, fabricated in Photoresist 2 (100 % LP, 10000 μm/s); **b** Scattering spectra corresponding to highlighted micropillars (Scale bar represents 100 μm).

Supplementary Table S13. Interparticle distance values calculated for Photoresist 2, using Eq.2 and the λ_{max} values measured upon varying both SL and HD parameters in micropillar array (100% LP, 10000 μm/s).

SL (μm)	HD (μm)	λ_{max} (nm)	d_{int} (nm)
0.1	0.1	719	246
0.2	0.2	557	191
0.3	0.3	479	164
0.4	0.4	434	148
0.5	0.5	397	136

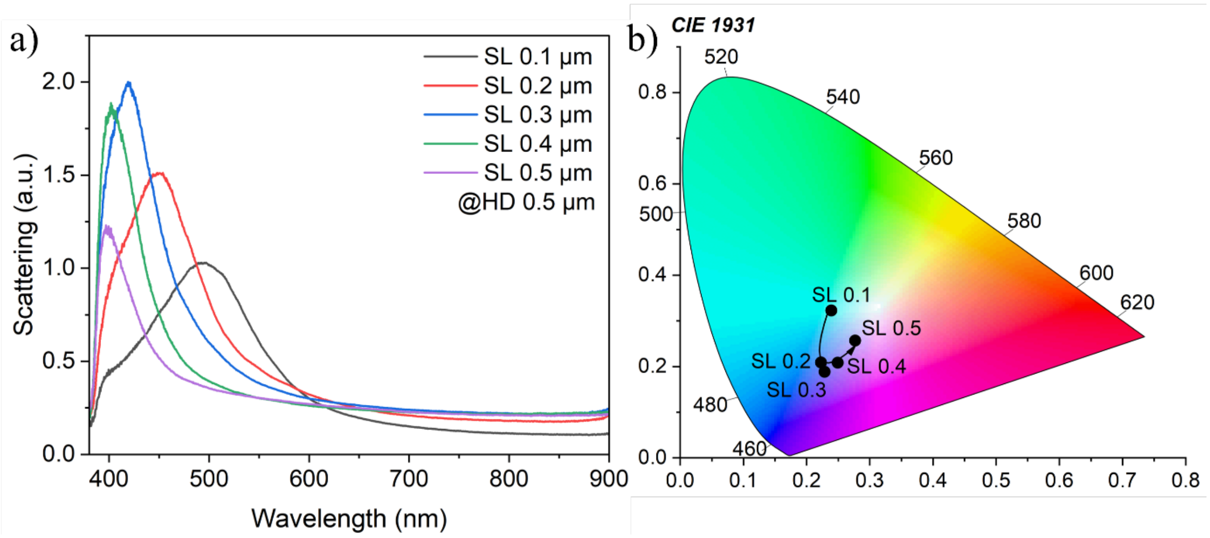

Supplementary Figure S10. a Scattering spectra of hydrated micropillar array fabricated in Photoresist 2, varying SL = 0.1 – 0.5 μm at constant HD = 0.5 μm (100% LP, 10000 μm/s); **b** corresponding CIE diagram.

Supplementary Table S14. λ_{max} values measured upon varying SL = 0.1 – 0.5 µm at constant HD = 0.5 µm in Photoresist 2 5 x 5 micropillar array (100% LP, 10000 µm/s).

SL (µm)	HD (µm)	λ_{max} (nm)
0.1	0.5	492
0.2	0.5	454
0.3	0.5	419
0.4	0.5	402
0.5	0.5	397

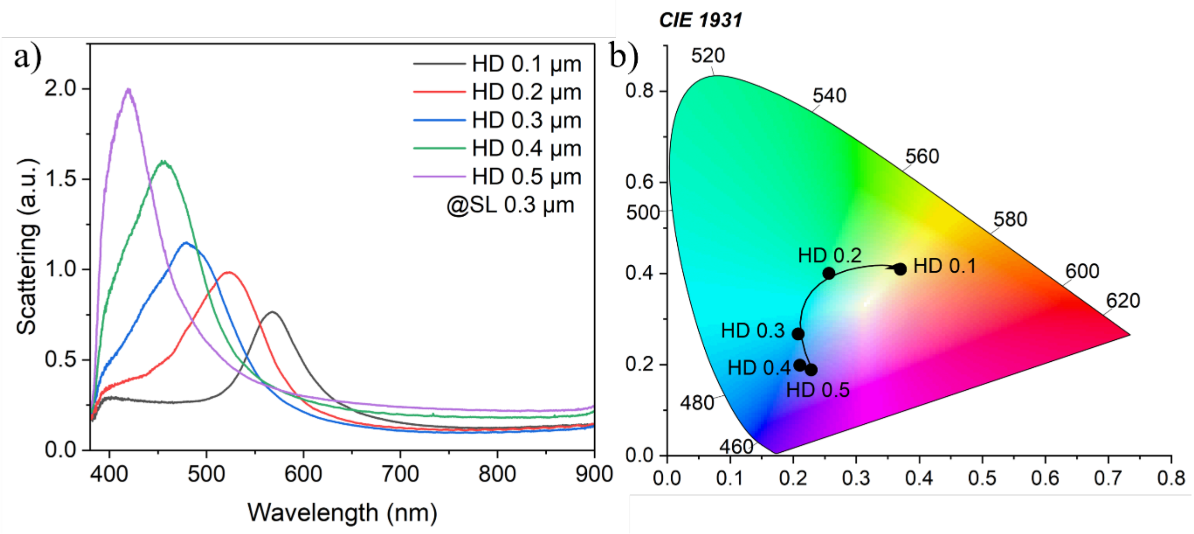

Supplementary Figure S11. a Scattering spectra of hydrated micropillar array fabricated in Photoresist 2, varying HD = 0.1 – 0.5 µm at constant SL = 0.3 µm (100% LP, 10000 µm/s); **b** corresponding CIE diagram.

Supplementary Table S15. λ_{max} values measured upon varying HD = 0.1 – 0.5 µm at constant SL = 0.3 µm in Photoresist 2 (100% LP, 10000 µm/s).

SL (µm)	HD (µm)	λ_{max} (nm)
0.3	0.1	567
0.3	0.2	525
0.3	0.3	479
0.3	0.4	454
0.3	0.5	419

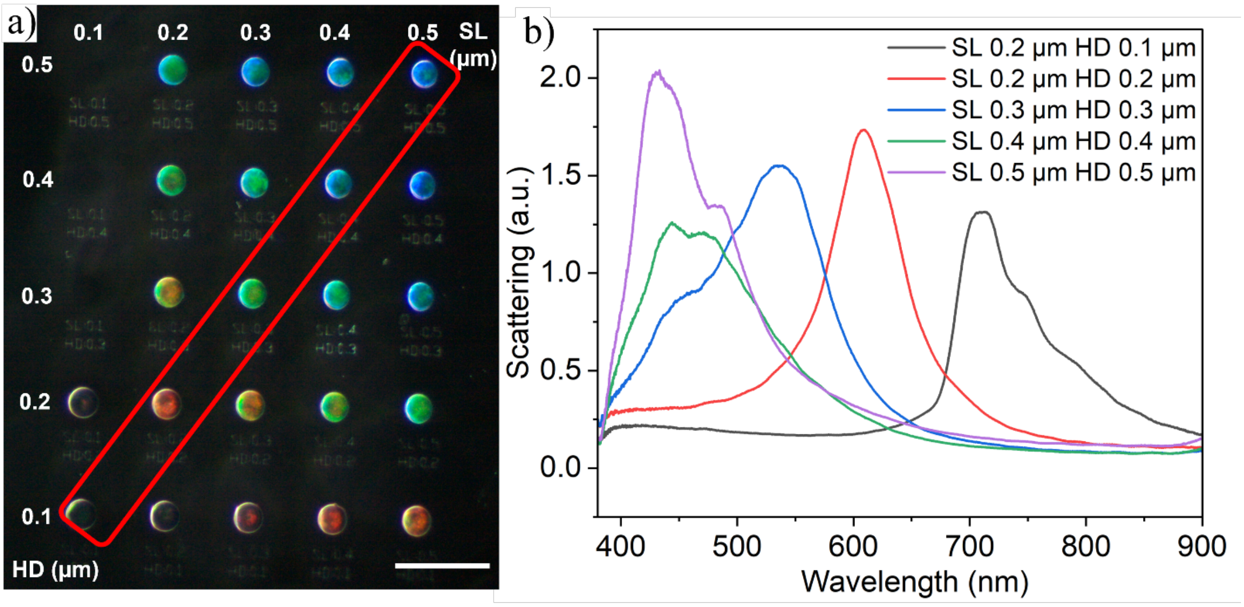

Supplementary Figure S12. a Dark field optical microscope image of 5 x 5 micropillar array, varying both slicing and hatching distance from 0.1 to 0.5 µm, fabricated in Photoresist 3 (90% LP, 10000 µm/s) (Scale bar represents 100 µm); **b** Scattering spectra corresponding to highlighted micropillars.

Supplementary Table S16. Interparticle distance values calculated for Photoresist 3, using Eq. 2 and λ_{max} values measured upon varying SL and HD parameters of micropillar array (90% LP , 10000 µm/s).

SL (µm)	HD (µm)	λ_{max} (nm)	d_{int} (nm)
0.2	0.2	609	208
0.3	0.3	535	183
0.4	0.4	444	152
0.5	0.5	433	148

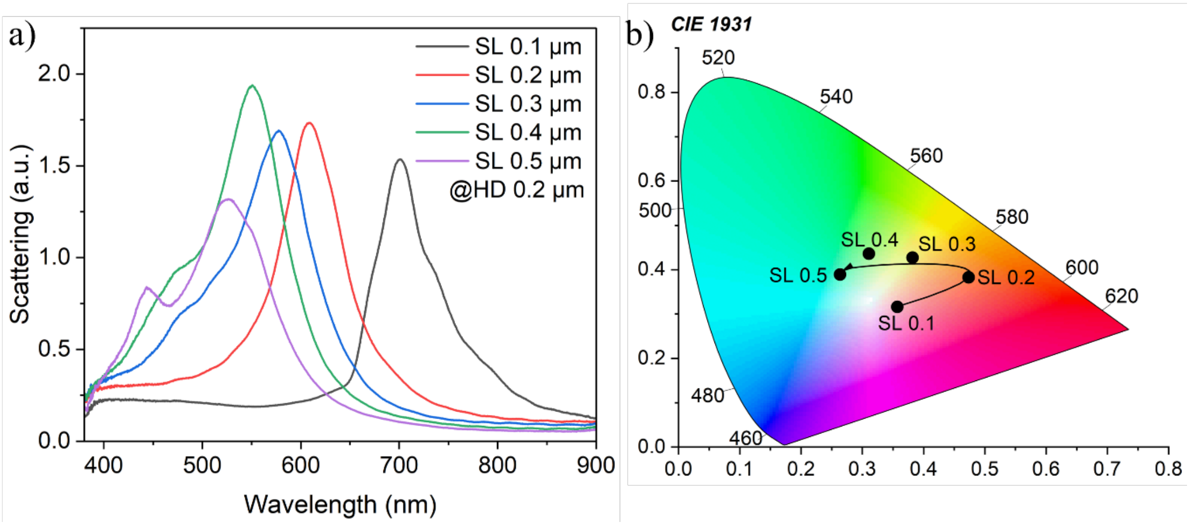

Supplementary Figure S13 a Scattering spectra of hydrated micropillar array fabricated in Photoresist 3, varying SL = 0.1 – 0.5 µm at constant HD = 0.2 µm (90% LP, 10000 µm/s) and **b** corresponding CIE diagram.

Supplementary Table S17. λ_{max} values measured upon varying SL = 0.1 – 0.5 µm at constant HD = 0.2 µm in Photoresist 3 micropillar array (90% LP, 10000 µm/s).

SL (µm)	HD (µm)	λ_{max} (nm)
0.1	0.2	701
0.2	0.2	609
0.3	0.2	578
0.4	0.2	551
0.5	0.2	526

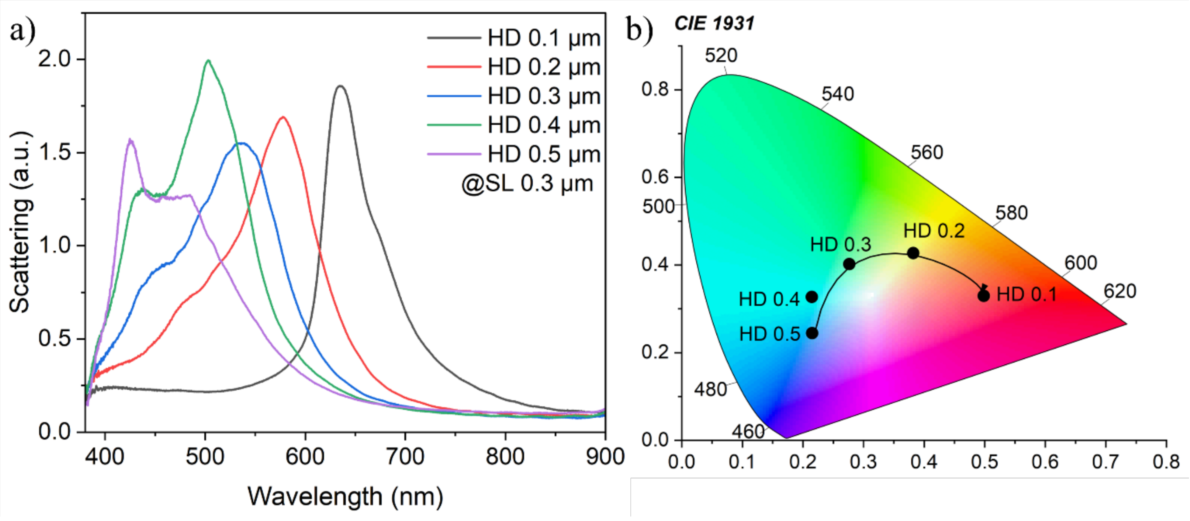

Supplementary Figure S14. a Scattering spectra of hydrated micropillar array fabricated in Photoresist 3, varying HD = 0.1 – 0.5 µm at constant SL = 0.3 µm (90% LP, 10000 µm/s) and **b** corresponding CIE diagram.

Supplementary Table S18. λ_{max} values measured upon varying HD = 0.1 – 0.5 µm at constant SL = 0.3 µm in Photoresist 3 micropillar array (90% LP, 10000 µm/s).

SL (µm)	HD (µm)	λ_{max} (nm)
0.3	0.1	636
0.3	0.2	578
0.3	0.3	536
0.3	0.4	503
0.3	0.5	425

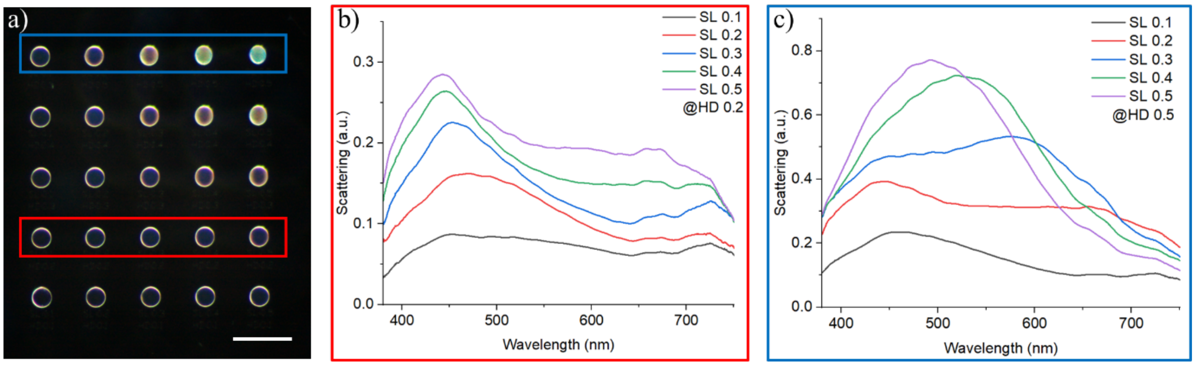

Supplementary Figure S15. a Dark field optical microscope images of a 5 x 5 micropillar array in DI water, varying both slicing and hatching distances from 0.1 to 0.5 µm, fabricated with a 40 wt% PNP1 photoresist (top, 100% LP, 5000 µm/s) (Scale bar represents 100 µm). Corresponding scattering spectra for variation of SL = 0.1 - 0.5 µm at constant HD = 0.2 µm (**b**) and HD = 0.5 µm (**c**). All spectra were smoothed using adjacent averaging.

Supplementary Table S19. AFM height measurements of a micro-cube array (10 x 10 x 3 µm) fabricated in Photoresist 1 (60% LP, 10000 µm/s), varying HD and SL (µm). Measurements performed in dry state.

(µm)	HD 0.1	HD 0.2	HD 0.3
**SL 0.1**	2.24 ± 0.02	1.99 ± 0.02	1.72 ± 0.04
**SL 0.2**	1.92 ± 0.02	1.52 ± 0.02	0.99 ± 0.04
**SL 0.3**	1.89 ± 0.02	1.39 ± 0.05	0.93 ± 0.06

Supplementary Table S20. AFM height measurements of a micro-cube array (10 x 10 x 3 µm) fabricated in Photoresist 1 (60% LP, 10000 µm/s), varying HD and SL (µm). Measurements performed in DI water.

(µm)	HD 0.1	HD 0.2	HD 0.3
**SL 0.1**	2.60 ± 0.02	2.38 ± 0.05	2.11 ± 0.005
**SL 0.2**	2.29 ± 0.03	1.9 ± 0.02	1.29 ± 0.05
**SL 0.3**	2.32 ± 0.02	1.73 ± 0.05	1.09 ± 0.09

Supplementary Table S21. Height difference percentages between AFM height measurements in the dry and hydrated state (DI Water), as reported above, for a 3x3 microcube array fabricated with Photoresist 1 (60% LP, 10000 µm/s) at varying slicing and hatching parameters.

(µm)	HD 0.1	HD 0.2	HD 0.3
**SL 0.1**	16.4	19.6	22.7
**SL 0.2**	19.4	25.7	29.5
**SL 0.3**	22.7	24.8	17.6

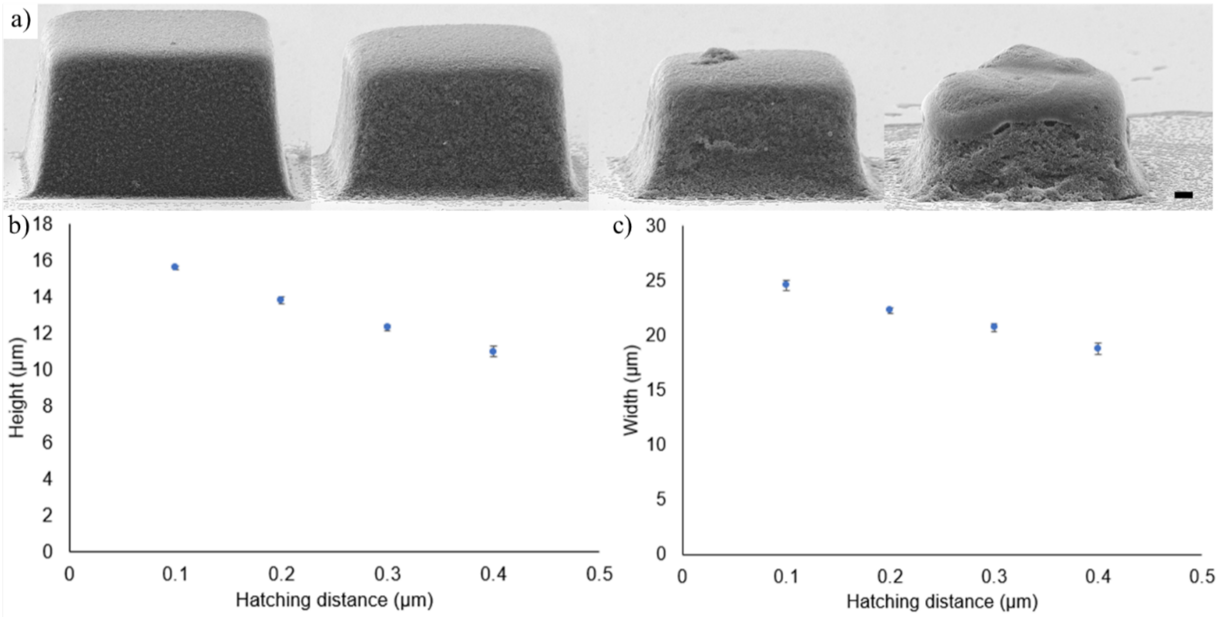

Supplementary Figure S16. a SEM images of a cube array 30 x 30 x 20 µm, fabricated with Photoresist 3 at 70% LP, 10000 µm/s with varying HD = 0.1 - 0.4 µm at constant SL = 0.3 µm, imaged at 80º tilt (scale bar represents 2 µm). **b** Cube height variation versus hatching distance. **c** Cube width variation versus hatching distance.

Supplementary Table S22. SEM Height measurements of cube array 30 x 30 x 20 µm, fabricated with Photoresist 3 at 70% LP, 10000 µm/s, with varying HD = 0.1 - 0.4 µm at constant SL = 0.3 µm (N = 5).

SL (µm)	HD (µm)	Height (µm)	Width (µm)
0.3	0.1	15.6 ± 0.1	24.6 ± 0.4
0.3	0.2	13.8 ± 0.2	22.3 ± 0.3
0.3	0.3	12.3 ± 0.1	20.7 ± 0.4
0.3	0.4	11 ± 0.3	18.8 ± 0.5

Supplementary Table S23. SEM Height measurements of cube array 30 x 30 x 20 µm, fabricated with Photoresist 3 at 70% LP, 10000 µm/s, with varying SL = 0.1 - 1 µm at constant HD = 0.2 µm (N = 5).

SL (µm)	HD (µm)	Height (µm)	Width (µm)
0.1	0.2	15.6 ± 0.1	25.8 ± 0.3
0.2	0.2	14.6 ± 0.2	24.3 ± 0.3
0.3	0.2	14.2 ± 0.1	22.5 ± 0.2
0.4	0.2	13.4 ± 0.2	20.1 ± 0.6
0.5	0.2	12.6 ± 0.1	19.3 ± 0.5
0.6	0.2	11.4 ± 0.2	18.1 ± 0.6
0.7	0.2	10.7 ± 0.2	17.8 ± 0.5
0.8	0.2	9.8 ± 0.3	16.4 ± 0.8
0.9	0.2	8.8 ± 0.3	15.1 ± 1.1
1	0.2	8.3 ± 0.2	15.6 ± 1.4

Supplementary Table S24. Simulated reflected wavelengths based on varying only nanoparticle diameter.

Diameter (nm)	Wavelength (nm)
80	525.5
100	528.8
120	528.8
140	532.1
160	538.8

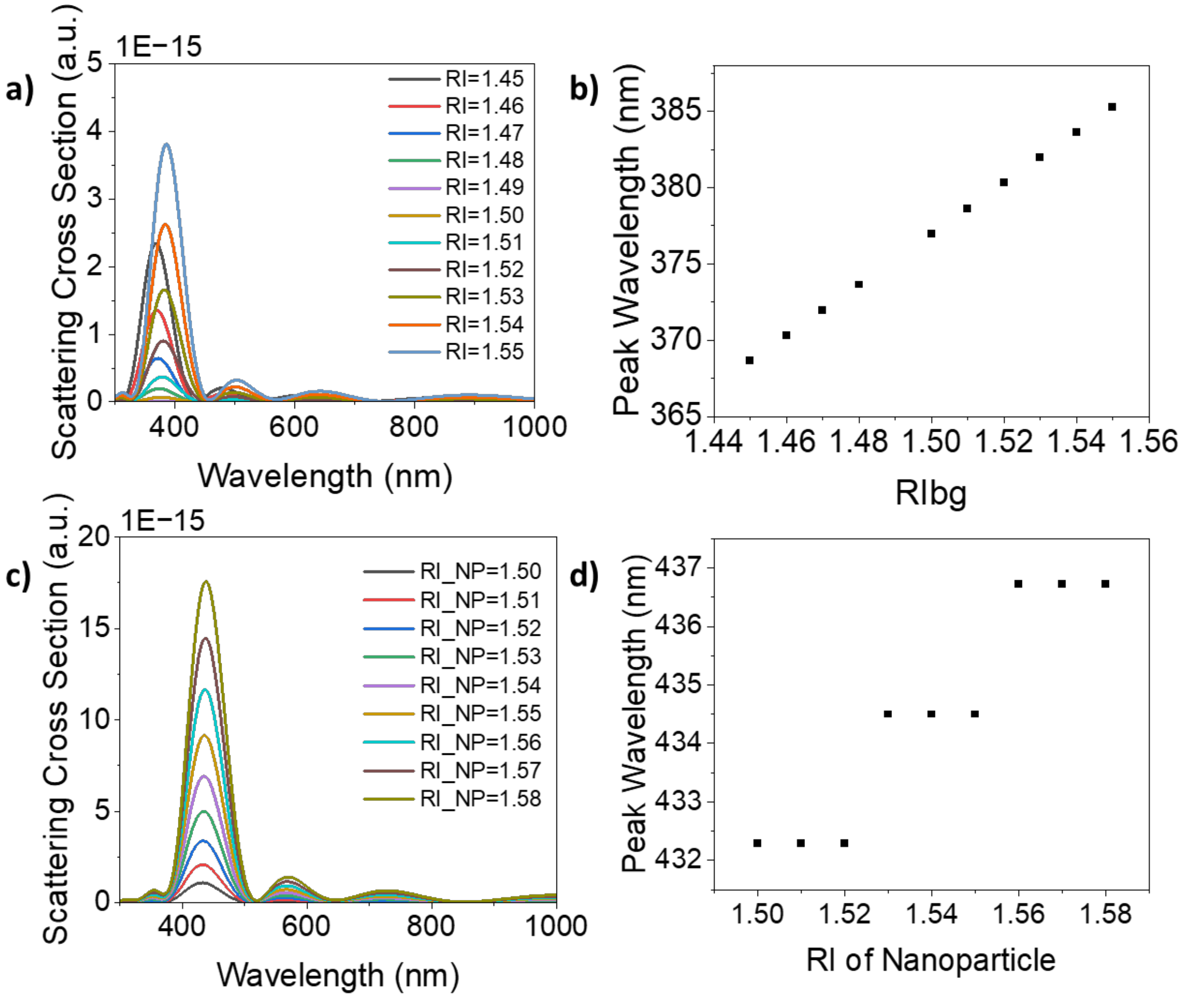

Supplementary Figure S17. FDTD simulations. **a** Simulated scattering cross section of FCC (111) structure upon changing refraction index of background polymer (RIbg) from 1.45 to 1.55. Diameter of nanoparticles fixed at 117 nm, 50 vol% nanoparticles are embedded in the polymer with 15% structure expansion in water (D = 153.4 nm, d_{int} = 125.2 nm, RI = 1.49). **b** Corresponding peak wavelengths of spectra in **a**. **c** Simulated scattering cross section of the FCC (111) structure upon varying refractive index of nanoparticles (RI) from 1.50 to 1.58. Diameter of nanoparticles fixed at 130 nm, 50 vol% nanoparticles constitute the microstructure within polymer with consideration of 20% expansion of nanoparticle-polymer system in water (D = 177.8 nm, d_{int} = 145.2 nm, RIbg = 1.474). **d** Corresponding peak wavelengths of spectra in **c**.

Supplementary Table S25. D, d_{int} and RIbg values corresponding to scattering cross section of expanded FCC (111) structure (30 vol% nanoparticles) with increasing expansion ratios in water (d_{pnp}= 117 nm, RI = 1.49), shown in Fig. 4. 3g. RIbg values estimated using Maxwell-Garnett approx.

Expansion %	D (nm)	d_{int} (nm)	RIbg
**10%**	173.9	143.0	1.475
**20%**	189.7	155.0	1.459
**30%**	205.6	167.8	1.443
**40%**	221.4	180.7	1.427
**50%**	237.2	194.0	1.411

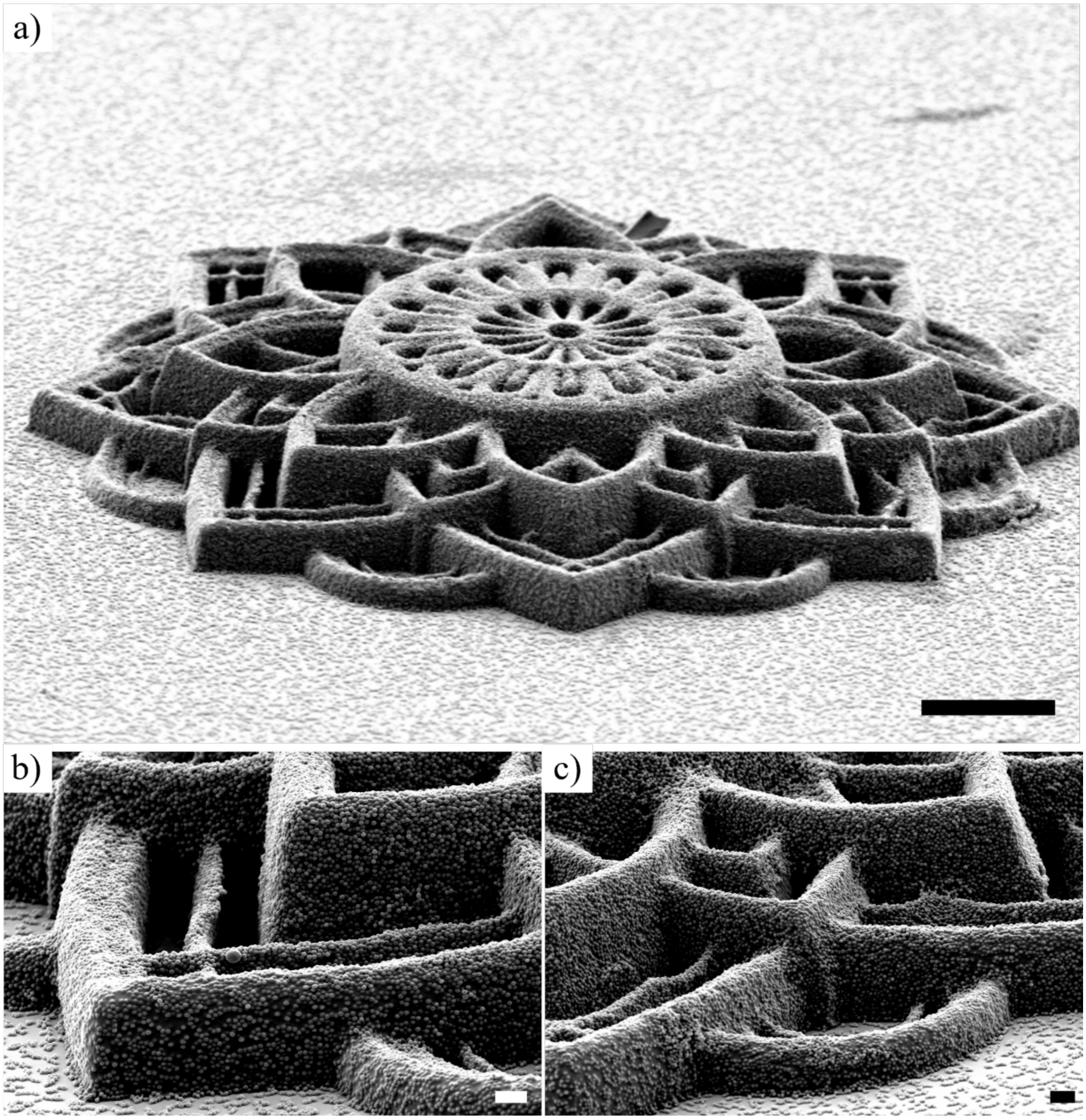

Supplementary Figure S18. SEM images of mandala design. **a** mandala design 1 (scale bar represents 10 µm) fabricated with Photoresist 3 (100% LP, 7000 µm/s) with **b, c** magnified details of features (scale bar represents 1 µm). Sample imaged at 70° tilt.

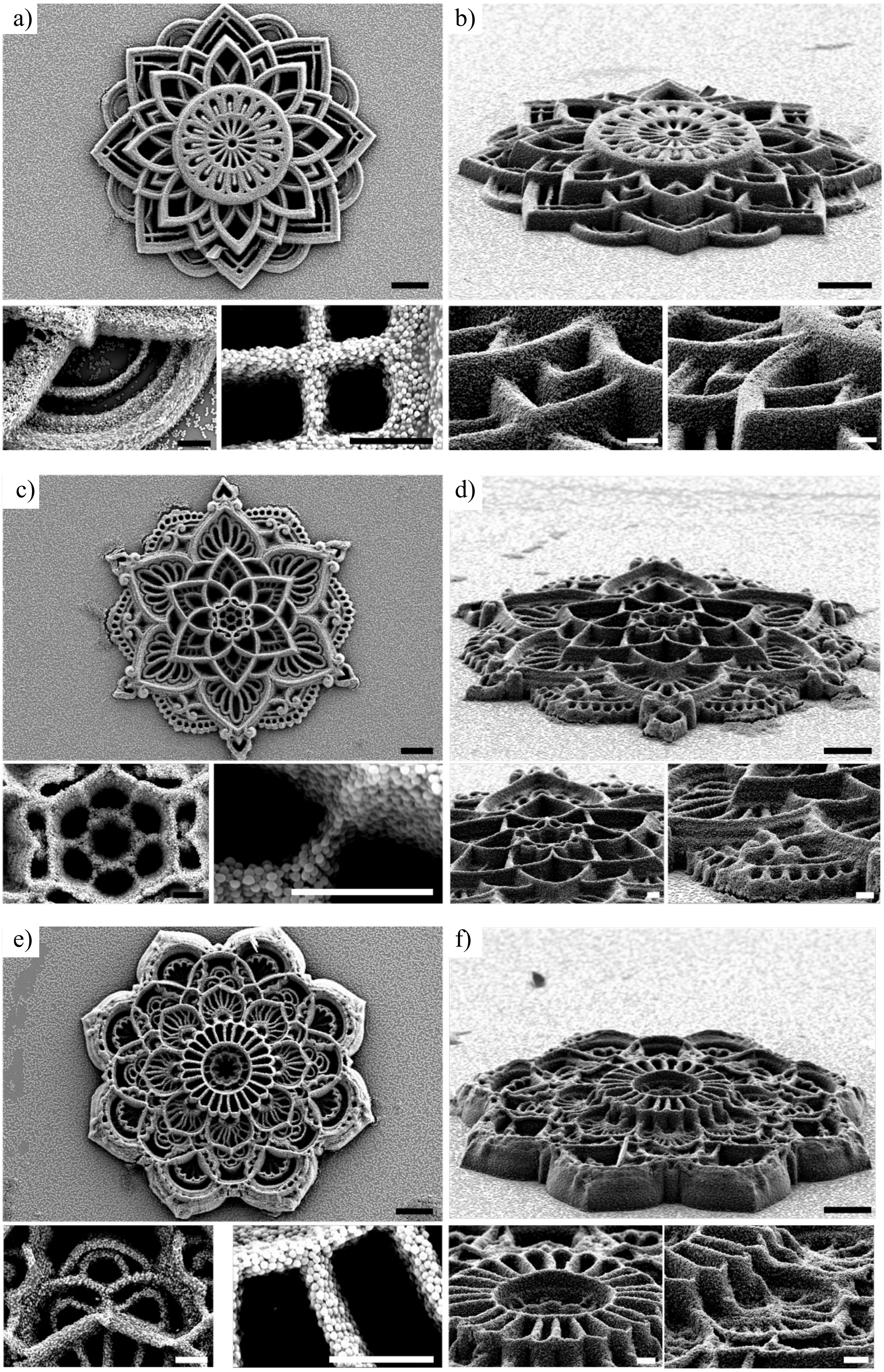

Supplementary Figure S19. SEM images recorded top down (left) and at 70° tilt angle perspective (right) for composite microstructures fabricated from Photoresist 3; **a-b** mandala design 1, **c-d** mandala design 2, **e-f** mandala design 3. Scale bar represents 10 µm in the images showing the full mandala structures, and 2 µm in the high-magnification detail images.

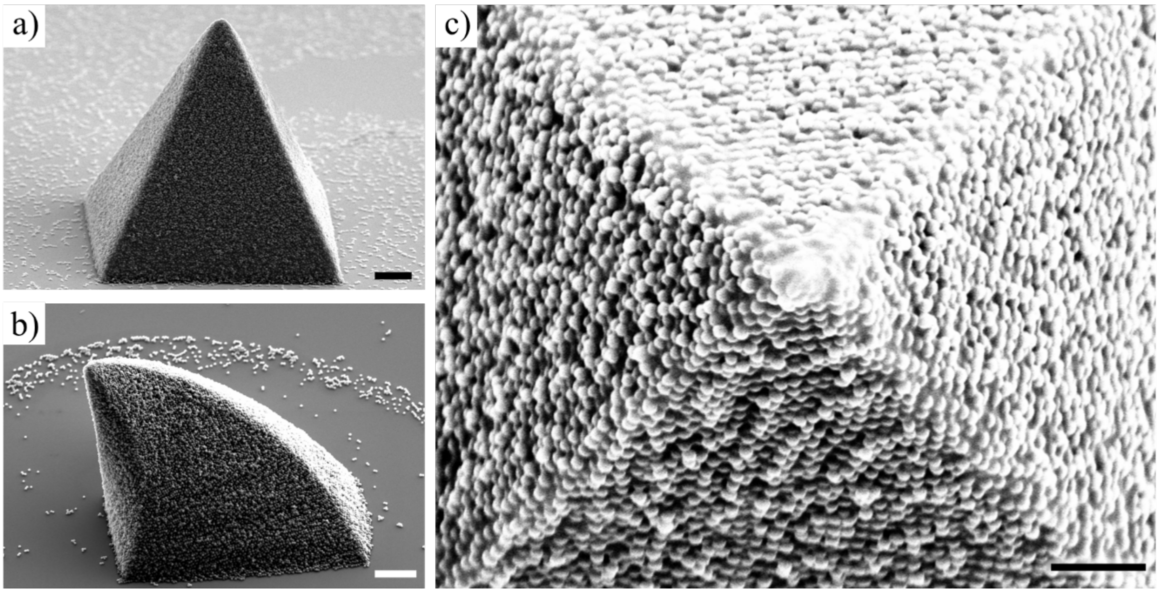

Supplementary Figure S20. SEM images of **a** square pyramid, of 20 µm width and 24 µm height (scale bar represents 3 µm, 70° tilt angle) with **c** top-down detail (scale bar represents 1 µm), and a **b** twisted pyramid of 25 µm in width and 20 µm in height (scale bar represents 3 µm, 40° tilt angle). All structures were fabricated with Photoresist 3 (100% LP, 7000 µm/s).

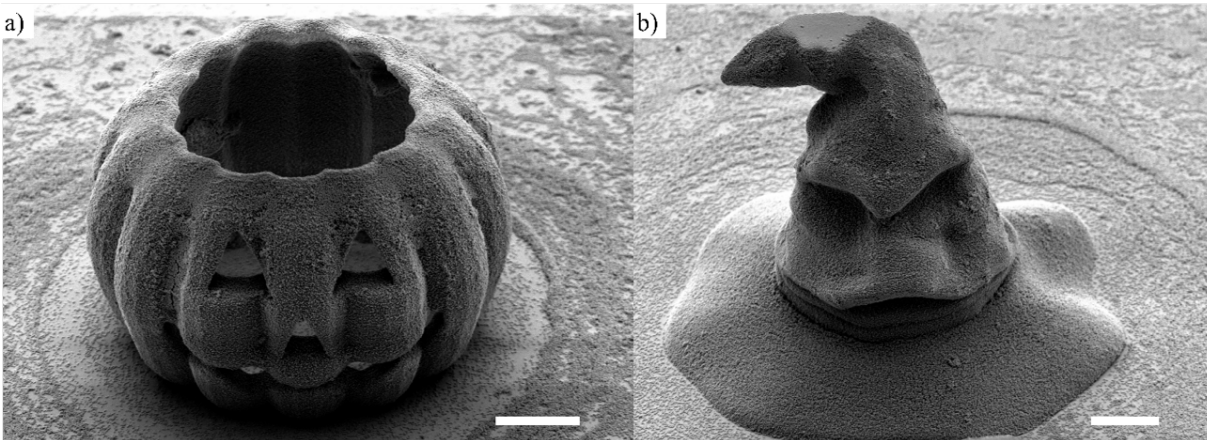

Supplementary Figure S21. SEM images taken at a 45° tilt of composite microstructures fabricated from Photoresist 1. **a** Pumpkin design (h = 40 µm, wall thickness = 2 µm) (85% LP 10000 µm/s). **b** Sorting hat design (h = 30 µm) (100% LP 10000 µm/s). Scale bars represent 10 µm.

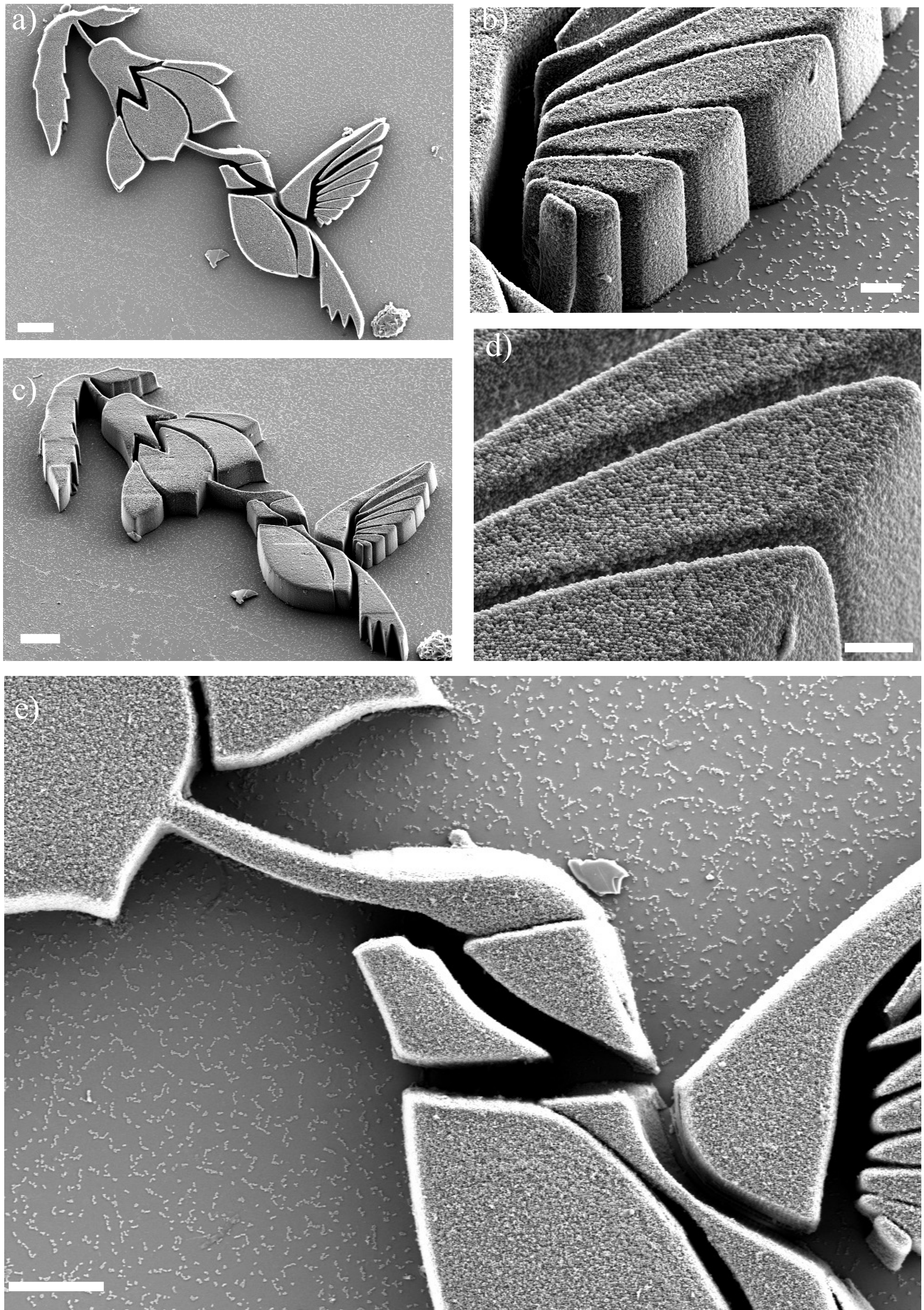

Supplementary Figure S22. SEM images of hummingbird design, showing areas with different fabrication parameters. Scale bar represents 20 µm in **a** (top down), 4 µm in **b** (40 ° tilt), 20 µm in **c** (40 ° tilt), 3 µm in **d** (40 ° tilt), and 10 µm in **e** (top down).

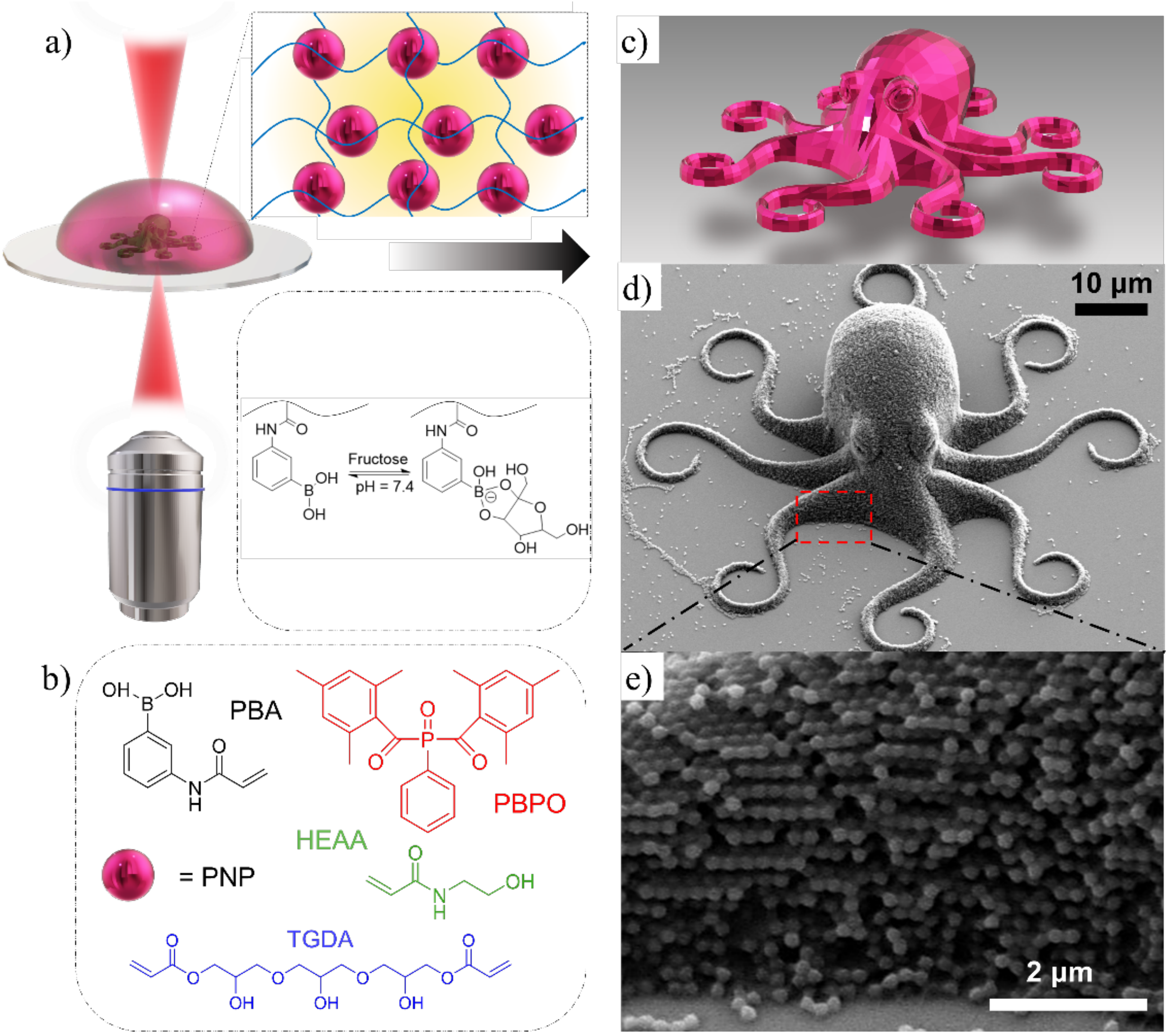

Supplementary Figure S23. a Schematic representation of the DLW process using Photoresist 4, showing binding to fructose at pH = 7.4; **b** Composition of Photoresist 4 showing the chemical structures of TGA, HEAA, PBA, PBPO and PNP (55 wt%, d_{SEM} = 173 ± 7 nm; d_{hyd} = 241 ± 5 nm); Detailed composition available in **Supplementary Table S5**. **c** 3D design of an octopus structure; **d** SEM images of the corresponding octopus microstructure fabricated in Photoresist 4, 95% LP, 10000 µm/s; **e** magnified SEM image of structure details.

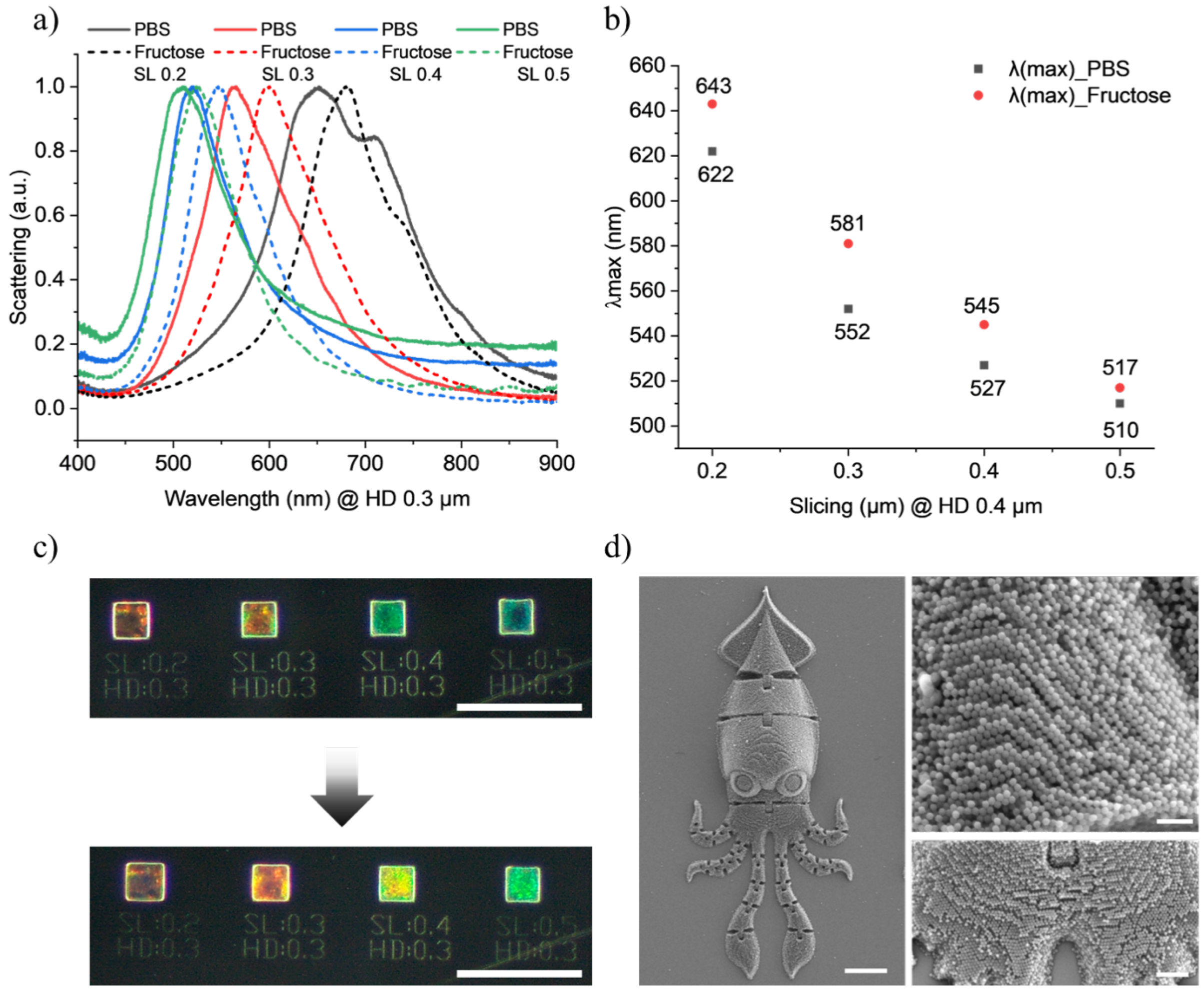

Supplementary Figure S24. Sugar-responsive polymer nanocomposite (SI Table 5). **a** Superimposed normalised scattering spectra in Phosphate Buffer Solution (PBS) and fructose (100 mM) of cuboid structures SL= 0.2 - 0.5 µm at HD = 0.3 µm (85% LP, 10000 µm/s). **b** λ_{max} vs. Slicing distance graph showing λ_{max} changes from PBS to fructose (100 mM). **c** Corresponding dark field microscopy images showing observed colors in PBS (top) vs. fructose (100 mM) (bottom). Scale bars represent 100 µm. **d** SEM images of sugar-responsive squid microstructure (100% LP, 10000 µm/s%) (left scale bar: 10 µm; top right scale bar: 1 µm; bottom right scale bar: 2 µm).
